# Nuclear RNA-related processes modulate the assembly of cytoplasmic RNA granules

**DOI:** 10.1093/nar/gkae119

**Published:** 2024-02-16

**Authors:** Mor Angel, Eden Fleshler, Mohammad Khaled Atrash, Noa Kinor, Jennifer I C Benichou, Yaron Shav-Tal

**Affiliations:** The Mina & Everard Goodman Faculty of Life Sciences and Institute of Nanotechnology, Bar-Ilan University, Ramat Gan 5290002, Israel; The Mina & Everard Goodman Faculty of Life Sciences and Institute of Nanotechnology, Bar-Ilan University, Ramat Gan 5290002, Israel; The Mina & Everard Goodman Faculty of Life Sciences and Institute of Nanotechnology, Bar-Ilan University, Ramat Gan 5290002, Israel; The Mina & Everard Goodman Faculty of Life Sciences and Institute of Nanotechnology, Bar-Ilan University, Ramat Gan 5290002, Israel; The Mina & Everard Goodman Faculty of Life Sciences and Institute of Nanotechnology, Bar-Ilan University, Ramat Gan 5290002, Israel; The Mina & Everard Goodman Faculty of Life Sciences and Institute of Nanotechnology, Bar-Ilan University, Ramat Gan 5290002, Israel

## Abstract

Stress granules (SGs) are cytoplasmic assemblies formed under various stress conditions as a consequence of translation arrest. SGs contain RNA-binding proteins, ribosomal subunits and messenger RNAs (mRNAs). It is well known that mRNAs contribute to SG formation; however, the connection between SG assembly and nuclear processes that involve mRNAs is not well established. Here, we examine the effects of inhibiting mRNA transcription, splicing and export on the assembly of SGs and the related cytoplasmic P body (PB). We demonstrate that inhibition of mRNA transcription, splicing and export reduces the formation of canonical SGs in a eukaryotic initiation factor 2α phosphorylation-independent manner, and alters PB size and quantity. We find that the splicing inhibitor madrasin promotes the assembly of stress-like granules. We show that the addition of synthetic mRNAs directly to the cytoplasm is sufficient for SG assembly, and that the assembly of these SGs requires the activation of stress-associated protein synthesis pathways. Moreover, we show that adding an excess of mRNA to cells that do not have active splicing, and therefore have low levels of cytoplasmic mRNAs, promotes SG formation under stress conditions. These findings emphasize the importance of the cytoplasmic abundance of newly transcribed mRNAs in the assembly of SGs.

## Graphical Abstract

**Graphical Abstract:**
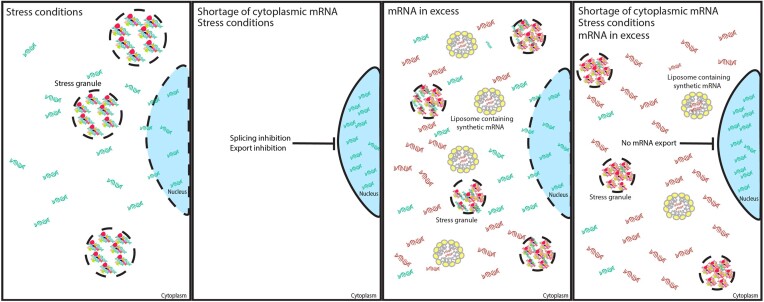

## Introduction

Messenger RNA (mRNA) stability and regulation of translation affect gene expression directly; newly transcribed mRNAs are bound by various RNA-binding proteins (RNBPs) to form ribonucleoprotein (RNP) granules, which influence RNA decay, localization and translation (1). There are various types of RNA granules [e.g. neuronal granules, processing bodies and stress granules (SGs)], classified mainly by their composition, cell localization and assumed function (2). SGs are cytoplasmic, membraneless, dynamic granules, characterized by their assembly under stress conditions, composed of RNBPs, translation initiation factors, poly(A)^+^ RNAs and 40S ribosome subunits (3,4). The composition of SGs may vary according to cell type, stress or disease state (5). For example, SGs induced by chronic starvation or sodium selenite lack eukaryotic initiation factor 3 (eIF3), and are therefore termed ‘noncanonical SGs’ (6,7).

Mechanistically, SGs are formed as a consequence of translation arrest (8,9). Overall, there are two main checkpoints of translation initiation: the first is the activation of eukaryotic initiation factor 2 (eIF2) and the assembly of the eIF2–GTP–tRNA_i_^Met^ ternary complex (10). The second is the recruitment of the cap-binding complex (eIF4F) to the 5′ end and to the 3′ poly(A) tail of the mRNA (11,12). The latter is regulated by the target of rapamycin (mTOR) complex (1). Thus, the inhibition of eIF2α by its phosphorylation and/or the eIF4F complex by mTOR leads to SG formation (1). eIF2α can be phosphorylated by four kinases in a process termed integrated stress response (ISR): general control nonderepressible 2 (GCN2), protein kinase R (PKR), eIF2α kinase (HRI) and PKR-like ER kinase (PERK). Each kinase is triggered by a different type of stress (7).

mRNAs are a key component of SGs; however, only recently studies have begun to reveal the role of mRNA in the assembly of SGs. It was widely demonstrated in previous studies (13,14) that RNBPs containing prion-like low-complexity intrinsically disordered protein regions promote the assembly of SGs, and lately it was discovered that RNAs play an important role in SG assembly as well, by mediating RNA–RNA and RNA–protein interactions (15,16). Furthermore, mRNA degradation leads to SG disassembly and limits their formation (17). The fact that multivalent RNA–RNA interactions can induce RNA oligomerization and phase separation, together with the ability of a single transcript to interact with different RNBPs simultaneously to form multiprotein complexes, makes RNA a suitable player in the process of SG assembly. Studies in yeast revealed that RNA alone is enough to trigger SG formation, yet in order to form canonical SGs, a variety of transcripts are needed (18,19).

Generally, SG formation is influenced by the assembly and disassembly of translating polysomes; namely, an increase of untranslated mRNAs favors SG assembly (20). It was demonstrated that transfecting mRNA upon polysome dissociation promotes the formation of SGs, while an increase of RNBPs in the cytoplasm can prevent SG formation by isolating the mRNAs. As for the role of nascent mRNAs in SG assembly, it was recently suggested that newly exported mRNAs might have an advantage in SG assembly (21). It was also shown that inhibition of mRNA synthesis by actinomycin D (ActD) or triptolide reduces SG formation induced by oxidative stress (20,21).

The importance of mRNAs in SG formation has come to light lately; however, the connection between SG formation and mRNA-related nuclear processes such as transcription, splicing and mRNA export is not well established. Specifically, it is unclear how inhibition of these processes would affect SG formation; for instance, less availability of mature mRNA in the cytoplasm may impair the process of SG assembly. Here, we examined the effects of transcription, splicing and export inhibition on the assembly and disassembly of SGs and other mRNA-containing granules. We found that transcription and splicing inhibition suppress the formation of SGs. Using a variety of pre-mRNA splicing inhibitors, we demonstrated that SG assembly is dramatically reduced, and was independent of eIF2α phosphorylation levels. Moreover, splicing inhibitors had an effect not only on SGs, but also on other RNA granules such as P bodies (PBs). Blocking the export of nascent transcripts to the cytoplasm, thereby reducing the levels of mature mRNAs in the cytoplasm, also reduced SG assembly. Consequently, we examined how the addition of synthetic mRNAs directly to the cytoplasm, bypassing the nucleus, would affect SG assembly. We found that these transcripts were sufficient for SG formation even without applying stress. Furthermore, in stressed cells, while splicing was inhibited, mRNA in excess promoted SG formation, emphasizing the importance of cytoplasmic mRNA abundance in the assembly of SGs.

## Materials and methods

### Cell culture and transfections

Human U2OS (ATCC), E3 and E6 cells (22) and GFP-IGF2BP3 expressing cells (23) were maintained in low-glucose Dulbecco’s modified Eagle medium (DMEM; 01-050A, Sartorius, Israel). A549 cells (gift from Amit Tzur, Bar-Ilan University, Israel) and U2OS ΔΔG3BP1/2 cells (gift from Nancy Kedersha and Pavel Ivanov, Brigham and Women’s Hospital and Harvard Medical School, Boston, MA) (8) were grown on high-glucose DMEM (41965039, Gibco, USA). All cells were supplemented with 10% fetal bovine serum (SV30160.03, HyClone Laboratories, Logan, UT).

The stable U2OS cell line expressing mCherry-TIA1 (23) was created using co-transfection of 10 μg mCherry-TIA1 plasmid (received from Eran Hornstein, Weizmann Institute of Science, Rehovot, Israel) and 500 ng puromycin resistance plasmid, using electroporation (Gene Pulser Xcell, Bio-Rad). After transfection, cells were selected with puromycin (1 μg/ml; Invivogen, San Diego, CA, ant-pr-1). For splicing inhibition, cells were treated with pladienolide B (PLB; 0.5 μM, Santa Cruz, 445493) for 6 or 24 h, with isoginkgetin (50–100 μM, EMD Millipore, purchased from Sigma, 416154) for 4 h or with madrasin (20–30 μM, Sigma, SML1409) for 4 h, all dissolved in dimethyl sulfoxide (DMSO) (Sigma, D2650). For transcription inhibition, cells were treated with ActD (5 μg/ml, Sigma, A9415) for 2.5 h, DRB (50 μg/ml, Sigma, D1916) for 2.5 h, α-amanitin (30 μg/ml, Sigma, A2263) for 8 h or triptolide (1 μM, Sigma, T3652) for 24 h. All sodium arsenite treatments (0.25 mM, Sigma, S7400) were applied 30 min before fixation. Washes were applied for 24 h with fresh medium.

Additional cell treatments include 100 μM vinorelbine (VRB; Sigma, V2264) dissolved in ultrapure water. Thapsigargin (1 μM, Sigma, T9033) dissolved in DMSO was applied for 1 h before fixation. Cycloheximide (100 μg/ml, Sigma, C4859) was applied for 30 min to madrasin- or VRB-treated cells. For histone deacetylation inhibition, cells were treated with trichostatin A (TSA; 150 nM, Sigma, T8852) for 7 h or with suberoylanilide hydroxamic acid (SAHA; 150 ng/ml, Sigma, SML0061) for 24 h. For verifying splicing inhibition, cells were induced to express the E6 gene by the addition of 3 μg/ml doxycycline (Sigma, D9891), overnight.

For mRNA transfections, cells were transfected with 0.25–1 μg mRNA using Lipofectamine 2000 (Invitrogen, 11668) and fixed after 1.5 h. ISRIB (5 μM, Sigma, SML0843), dissolved in DMSO, was applied for 3 h prior to transfection, 4.5 h overall. PERK was inhibited by GSK II (40 μM, EMD Millipore Corp., GSK2656157), PKR was inhibited by a PKR inhibitor (0.4 μM, Sigma, 527451) and GCN2 was inhibited using GCN2-IN (4 μM, MedChemExpress, HY100877); all were applied 3 h prior to transfection. Positive control for the PKR inhibitor was applied using 1 μg/ml Poly I:C (Sigma, P1530), transfected with Lipofectamine 2000 (Thermo Fisher, 11668027).

For mRNA export inhibition, cells were transfected with dominant-negative DEAD-box protein 5 (Dbp5-DN) (24) or GFP-2xGLFG/HoxA9 (25) for 24 h using Lipofectamine 2000 (Invitrogen, 11668; 1.6 μg plasmid), followed by sodium arsenite treatment (0.25 mM, Sigma, S7400) for 30 min. Negative control was an empty plasmid.

### Western blotting

Cells were washed in cold phosphate-buffered saline (PBS), and proteins were extracted using immunoprecipitation lysis buffer (Thermo Fisher Scientific, TS-87787) containing 10 mM sodium fluoride (Sigma, S7920), 10 mM sodium orthovanadate (Sigma, S6508), 1 mM protease inhibitor cocktail (Sigma, P8340) and 1 mM phenylmethylsulfonyl fluoride (Sigma, P7626). The samples were then placed on ice for 20–25 min. The resulting lysate was centrifuged at 20 817 × *g* for 10 min at 4°C. Then, 20–40 μg/μl of protein per lane was run on sodium dodecyl sulfate–polyacrylamide gels and transferred to a nitrocellulose membrane (0.45 μm; Bio-Rad, 1620115). The membrane was blocked in 5% bovine serum albumin (BSA; Fisher Scientific, 02160006080) for 1 h at room temperature (RT) or overnight at 4°C, and then probed with a primary antibody for 2 h at RT or overnight at 4°C, followed by incubation with an HRP-conjugated goat anti-rabbit IgG secondary antibody (Abcam, ab7090) for 1 h at RT. Bands were detected using an enhanced chemiluminescence kit (Pierce, TS-34580). Primary antibodies used were rabbit anti-eIF2α (Cell Signaling Technology, 9722; 1:1000) and rabbit anti-phospho-eIF2α (Abcam, ab32157; 1:1000). Rabbit anti-α-tubulin (Abcam, ab4074; 1:000) was used for loading control. To normalize the different bands to control conditions and obtain a relative value, the ratio of phosphorylated eIF2α to eIF2α was calculated in the untreated group. Each treatment group was normalized to this value by taking the ratio of phosphorylated eIF2α to eIF2α and dividing it by the normalizing value from the control group. The standard deviation was also calculated. Quantification of band intensity from the blots was done using ImageJ software.

### mRNA synthesis

For *in vitro* transcription of mRNAs, plasmids encoding GFP (pGEM-GFP plasmid) or T-cell receptor (TCR) α or β chains were obtained from Cyrille Cohen, Bar-Ilan University, Ramat Gan, Israel (26). GFP-C1 plasmid (Clontech) served as a template for creating a shortened GFP RNA using reverse transcription polymerase chain reaction (RT-PCR) with the primers (forward: 5′-agaggatccaccggtcgccacca-3′; reverse: 5′-catactgtacatgggccagggcacgg) generating a 250-bp product that was ligated to the pGEM-GFP plasmid cut with AgeI and BsRGI. Plasmids were purified using a PCR purification kit (Geneaid, DFH100) and linearized using the SpeI restriction enzyme (New England Biolabs, R3133S). Linearized plasmids were used as templates for the *in vitro* transcription reaction using the AmpliCap-Max T7 High Yield Message Marker Kit (CELLSCRIPT, LLC, C-ACM04037). mRNAs were purified by RNA clean-up and concentration kit (Norgen Biotek Corp., 23600). mRNA concentration was determined by measuring the absorbance at 260 nm.

### Immunofluorescence

Cells were grown on coverslips in a 12-well plate and fixed in 4% paraformaldehyde (PFA) for 20 min. Cells were permeabilized in 0.5% Triton X-100 for 2 min, and blocking was applied using 5% BSA fraction V (MP Biomedicals, 160069). Then, cells were incubated with primary antibodies for 1 h, washed with 1× PBS, and incubated with secondary fluorescent antibodies. The nucleus was stained with Hoechst H33342 (1 μg/ml, Sigma, B2261) and coverslips were mounted in mounting medium (homemade). Primary antibodies: anti-G3BP1 (1:200, Abcam, ab56574), anti-TIA1 (1:100, Abcam, ab140595), anti-eIF4B (1:200, Abcam, ab68474), anti-eIF3b (1:150, Santa Cruz, sc-137214), anti-Caprin (1:200, Abcam, ab205377), anti-Dcp1a (1:250, Abcam, ab183709), anti-SF3B1 (1:200, Abcam, ab170854), anti-SC35 (1:500, Sigma, S4045), anti-SON (1:400, Sigma, HPA023535) and anti-RPS6 (1:200, Cell Signaling Technology, 5G10). For SF3B1 staining, cells were fixed in methanol by applying 100% cold methanol (DAEJUNG, 5558-4100) for 5 min, followed by 100% cold acetone (Bio-Lab, 00103100500) for 2 min. Secondary antibodies were purchased from Abcam [Alexa Fluor 488 goat anti-mouse (1:1000, ab150113), Alexa Fluor 488 goat anti rabbit (1:1000, ab150077), Cy3-Alexa Fluor goat anti-mouse (1:1000, ab97035), Cy7-Alexa Fluor goat anti-mouse (1:500, ab175738) and Cy5 donkey anti-goat (1:1000, ab6566)] and from Invitrogen [Alexa Fluor 647 anti-mouse (1:1000, A21235) and Alexa Fluor 647 anti-rabbit (1:1000, A31573)].

### Fluorescence *in situ* hybridization

Cells were grown on coverslips and fixed in 4% PFA for 20 min, and then kept in ethanol 70% (CARLO ERBA Reagents, 4146052) overnight at 4°C. Cells were washed with 1× PBS and treated for 2 min with 0.5% Triton X-100 (Sigma). Then, cells were washed twice with 1× PBS, incubated for 10 min in 15% formamide solution in a final concentration of SSC 4× (Bio-Lab, 001985232300) and hybridized overnight at 37°C in 15% formamide containing single-stranded DNA (ssDNA; Sigma, D7656), transfer RNA (tRNA; Roche, 10109541001) and fluorescently labeled oligo dT-Cy3 DNA probe (∼10 ng probe, 50-mer) for detecting poly(A) tails. The next day, cells were washed twice with 15% formamide for 15 min, and then washed for 1 h with 1× PBS. Immunofluorescence was performed after RNA fluorescence *in situ* hybridization (FISH). For exon/intron RNA FISH on E6 cells, the wash and hybridization buffer contained 40% formamide, ssDNA/tRNA and fluorescently labeled DNA probes to the intron or exon sequences (∼10 ng per coverslip) (22). For transcription site detection, E3 cells were hybridized with a probe to the MS2 region (27) (∼10 ng per coverslip), in 40% formamide solution. Formamide was purchased from Sigma (F7503) for FISH poly(A)^+^ or from Ambion (AM9432) for the intron/exon FISH. Single-molecule FISH experiments with Stellaris probes (Biosearch Technologies) were performed according to the manufacturer’s adherent cell protocol for MKI67 mRNA, as previously described (28,29).

### siRNA

Cells were transfected with small interfering RNAs (siRNAs; IDT, Israel) to knock down SF3B1 using Lipofectamine 2000 (Invitrogen, 11668). Arsenite treatment (0.25 mM, 30 min, Sigma, S7400) was applied 72 h after siRNA knockdown. Catalog numbers: 460262971, rArUrArUrUrGrArArGrCrArCrArGrA; 460262973, rGrArUrArCrGrGrUrGrArCrArUrUrCrArArU.

### Polysome profiling

U2OS cells were treated with PLB for 24 h and then incubated with 0.1 mg/ml cycloheximide for 30 min. Cells were harvested and washed once with ice-cold PBS containing 0.1 mg/ml cycloheximide, followed by two washes with ice-cold buffer containing 20 mM Tris (pH 7.6), 150 mM KCl, 10 mM MgCl_2_ and 10 mM cycloheximide. Whole-cell extracts were prepared using an ice-cooled Dounce homogenizer in the same buffer that also contains 0.5 M DTT (Sigma, D9779), 0.1% NP-40 (Sigma, I3021), 1 μl leupeptin (10 mg/ml, Sigma, L9783), 1 μl protease inhibitor cocktail (Roche 11836170001) and 1 μl of RNasin (Thermo Scientific, N8080119). The cell debris and nuclei from the lysate were pelleted at 14 000 rpm for 15 min and the supernatant was collected and fractionated on a 15–50% (w/v) sucrose gradient by centrifugation for 2 h at 39 000 rpm in a Beckman SW41 rotor at 4°C. The absorbance profile was measured at 254 nm and the positions of the 80S monosome and the polysomes are indicated.

### Nascent protein synthesis analysis

Following *in vitro* transcribed mRNA transfection, U2OS cells were incubated for 30 min with methionine-free medium (Gibco, USA) with 1% l-glutamine (Sigma, Biological Industries, 1639), and were fixed in 4% PFA. Nascent proteins were marked using the Click-iT HPG Alexa Flour 594 protein synthesis assay kit (Invitrogen, C10429), according to the manufacturer’s instructions, and were observed using fluorescence microscopy. Cycloheximide (100 μg/ml, Sigma, C4859) was applied 2 h before fixation.

### Nascent RNA synthesis analysis

U2OS cells were treated with splicing or transcription inhibitors, and nascent RNAs were labeled with the uridine analog 5-ethynyluridine (EU; 1 mM) using the Click-iT EU RNA Alexa Flour 488 imaging kit (Invitrogen, C10329) or the Click-iT Alexa Flour 594 kit (Invitrogen C10330). Cells were fixed by 4% PFA and EU-labeled nascent RNAs were observed using fluorescence microscopy. For RNA-labeled SG detection, cells were incubated with EU for 2 h in total, and were treated with PLB for 6 h or with arsenite for 1 h. Next, cells were fixed and immunofluorescence was performed, followed by the addition of the Click-iT reaction cocktail for 30 min.

### Imaging

Wide-field fluorescence images were obtained using the CellSens system based on an Olympus IX83 fully motorized inverted microscope (60× UPlanXApo objective, 1.42 NA) fitted with Prime BSI sCMOS (Teledyne) driven by the CellSens software. Colocalization analysis of two channels was performed using an ImageJ macro (Shav-Tal lab, Ramat Gan, Israel). SG area was analyzed using ImageJ. Live-cell imaging was carried out using the CellSens system with rapid wavelength switching. Cells were plated on glass-bottomed tissue culture plates (MatTek, Ashland, MA) in medium containing 10% fetal bovine serum. Imaging was carried out at 37°C, using an incubator that includes temperature and CO_2_ control (Life Imaging Services, Reinach, Switzerland). Live-cell movies were edited by ImageJ.

### Data analysis, statistics and quantifications

Experiments presented were repeated at least three times. Statistical analyses were performed using R statistical software (R Core Team, 2021. *R: A Language and Environment for Statistical Computing*. R Foundation for Statistical Computing, Vienna, Austria, https://www.R-project.org/) or by GraphPad Prism version 6.04 for Windows.

For counting the number of SG-positive cells in a cell population, SGs were counted manually with at least 100 cells per treatment, using ImageJ software (*n* = 3). For quantification of SG-positive cells, data were analyzed with independent sample *t*-tests (in the case of two groups) or with a one-way analysis of variance (ANOVA), followed by Tukey’s post hoc analysis. Treatment groups for which all values were constant (0% or 100% cells) were analyzed separately from other treatments using one-sample *t*-tests against a constant mean value of 0 or 100, respectively. Finally, *P*-values were adjusted for multiple comparisons with the Benjamini–Hochberg (false discovery rate) procedure.

For the analysis of granule size (SGs, PBs or nuclear speckles), data were log transformed (base 10). Treatment effect on granule size was tested with a one-way nested ANOVA (*n* = 3). The graph represents one experiment, and the statistical test for significance was carried out for all three experiments.

Statistical analysis for PBs per cell ratio and for band intensity western blotting data were log transformed (base 2) if needed to meet normality assumption. Then, data were tested with a one-way ANOVA, followed by Tukey’s post hoc analysis. Treatment groups for which all values were constant (0) were analyzed separately from other treatments using one-sample *t*-tests against a constant mean value of 0.

Cytoplasm to nucleus intensity ratio was quantified using ImageJ and data were analyzed by an independent sample *t*-test. More than 40 cells were quantified per treatment (*n* = 3). RNA poly(A)^+^ in the cytoplasm was quantified using ImageJ. In each field, the intensities of positive cells for Dbp5-DN were divided by the intensities of negative cells for Dbp5-DN, and a one-sample *t*-test analysis was carried out (>40 cells were quantified per treatment, *n* = 3).

Analysis of RNA FISH targeting the MKI mRNA was performed on U2OS cells. Images were acquired using wide-field microscopy (*z* = 51), and then images were deconvolved by Huygens Essential. Quantification of cytoplasmic MKI transcripts was performed using the Imaris Spot function to detect single mRNAs together with surface analysis on the Hoechst channel, for nuclei characterization. Single cells were obtained for analysis.

## Results

### Transcription inhibition has a negative effect on SG formation

**Figure 1.**
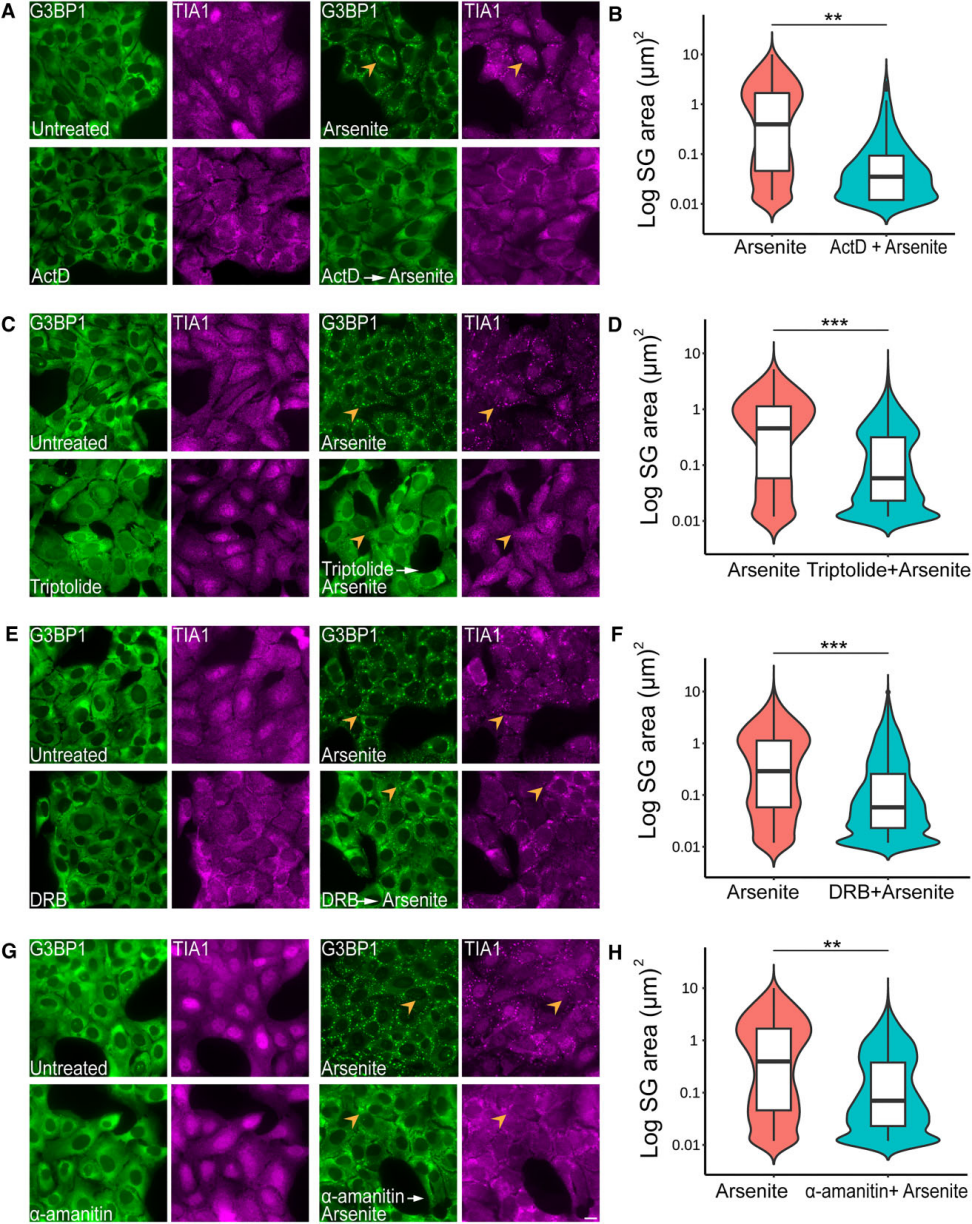
Transcription inhibition affects SG formation. U2OS cells were treated with (**A**, **B**) ActD (5 μg/ml; 2.5 h), (**C**, **D**) triptolide (1 μM; 24 h), (**E**, **F**) DRB (50 μg/ml; 2.5 h) and (**G**, **H**) α-amanitin (30 μg/ml; 8 h). Arsenite (0.25 mM) was added 30 min before fixation. Cells were stained with anti-G3BP1 as an SG marker (green) and anti-TIA1 (magenta). Scale bar = 20 μm. Orange arrowheads point to SG-positive cells. All data were analyzed using one-way nested ANOVA, and >1300 granules were quantified for each experiment per treatment (*n* = 3). The graph represents one experiment, and the statistical test for significance was carried for all three independent experiments (**P< 0.01, ***P< 0.001).

Inhibition of nascent mRNA synthesis by transcription inhibitors was previously shown to suppress SG formation suggesting that *de novo* RNA transcription plays a role in SG biogenesis by preventing RNA recruitment to SG nucleation sites, thus suppressing the assembly of SGs (20,21,30). Therefore, we first wanted to verify that treating cells with various transcription inhibitors, which act via different mechanisms, impairs SG formation. It was reported that the transcription inhibitor actinomycin D (ActD), which intercalates into the DNA, thus inhibiting RNA polymerases (RNAPs) I and II under certain concentrations (31,32), blocks the formation of SGs induced by arsenite (21). Under these conditions, relocalization of the shuttling RNBPs such as TIA1 and HuR from the nucleus to the cytoplasm occurs, and this was suggested to prevent SGs from forming while treating with arsenite (20). Indeed, treating cells with ActD suppressed SG formation significantly under arsenite conditions and the shuttling protein TIA1 was mostly observed in the cytoplasm (Figure 1A and B). Pretreating the cells with triptolide, which blocks transcription initiation (33), led to a significant decrease in SG size in comparison to arsenite treatment only, but did not change TIA1 localization (Figure 1C and D). Furthermore, treating the cells with 5,6-dichloro-1-β-d-ribofuranosylbenzimidazole (DRB) that affects RNAPII elongation (34), prior to arsenite treatment, also led to a significant reduction in SG size, in comparison to arsenite-treated cells (Figure 1E and F). Finally, treatment with α-amanitin, a cyclic peptide that inhibits RNAPII and RNAPIII (31), led to a significant decrease in SG size as well (Figure 1G and H). The reduction in SG size under these treatments, together with the fact that TIA1 did not change its localization under all transcription inhibitor conditions, led us to speculate that the effect of transcription inhibition on SG formation can be independent of the subcellular localization of RNBPs, and might be associated with the absence of mature untranslated mRNAs in the cytoplasm. Following our observations that transcription inhibition had a negative effect on SG formation, we asked whether enhanced transcription could impact SG formation. Cells were treated with TSA or with SAHA, both histone deacetylase inhibitors known to induce conditions of high activation of transcription, due to an increase in histone acetylation (35,36). These treatments did not affect SG formation (Supplementary Figure S1). This suggests that reduction in mRNA synthesis suppresses SG formation.

### Interfering with SF3B1 activity suppresses SG formation

**Figure 2.**
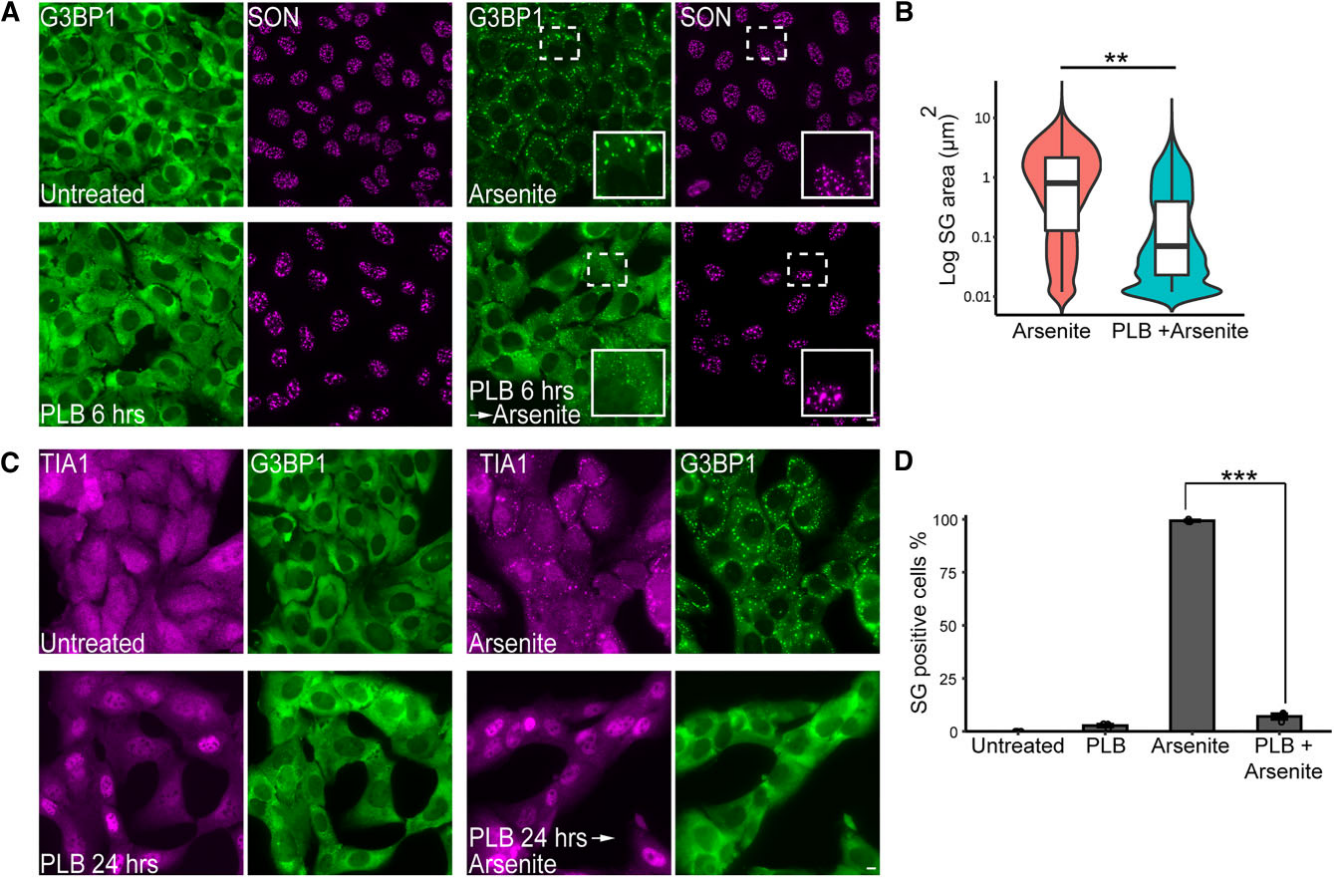
PLB splicing inhibitor suppresses SG formation. (**A**) U2OS cells were treated with PLB (0.5 μM; 6 h) and stained with anti-G3BP1 as an SG marker (green) and anti-SON (magenta) as a nuclear speckle marker. Arsenite (0.25 mM) was added 30 min before fixation. Enlargements of SGs and speckles are in the marked boxes. Scale bars = 10 μm. (**B**) Violin plot representing the area (log) of SGs, in cells treated as described in panel (A). Data were analyzed using one-way nested ANOVA. More than 5000 granules were quantified for each experiment per treatment (*n* = 3). The graph represents one experiment, and the statistical test for significance was carried for all three independent experiments (**P< 0.01). (**C**) U2OS cells were treated with PLB (0.5 μM; 24 h) and stained with anti-TIA1 (magenta) and anti-G3BP1 (green). Arsenite (0.25 mM) was added 30 min before fixation. (**D**) Quantification of the population of SG-positive U2OS cells treated as described in panel (C). Cells were counted in three independent experiments (*n* > 200 cells per treatment) and quantified by G3BP1 puncta. Data were analyzed with one-way ANOVA, followed by Tukey’s post hoc analysis (***P< 0.001). Bar graph illustrates the mean ± standard deviation.

**Figure 3.**
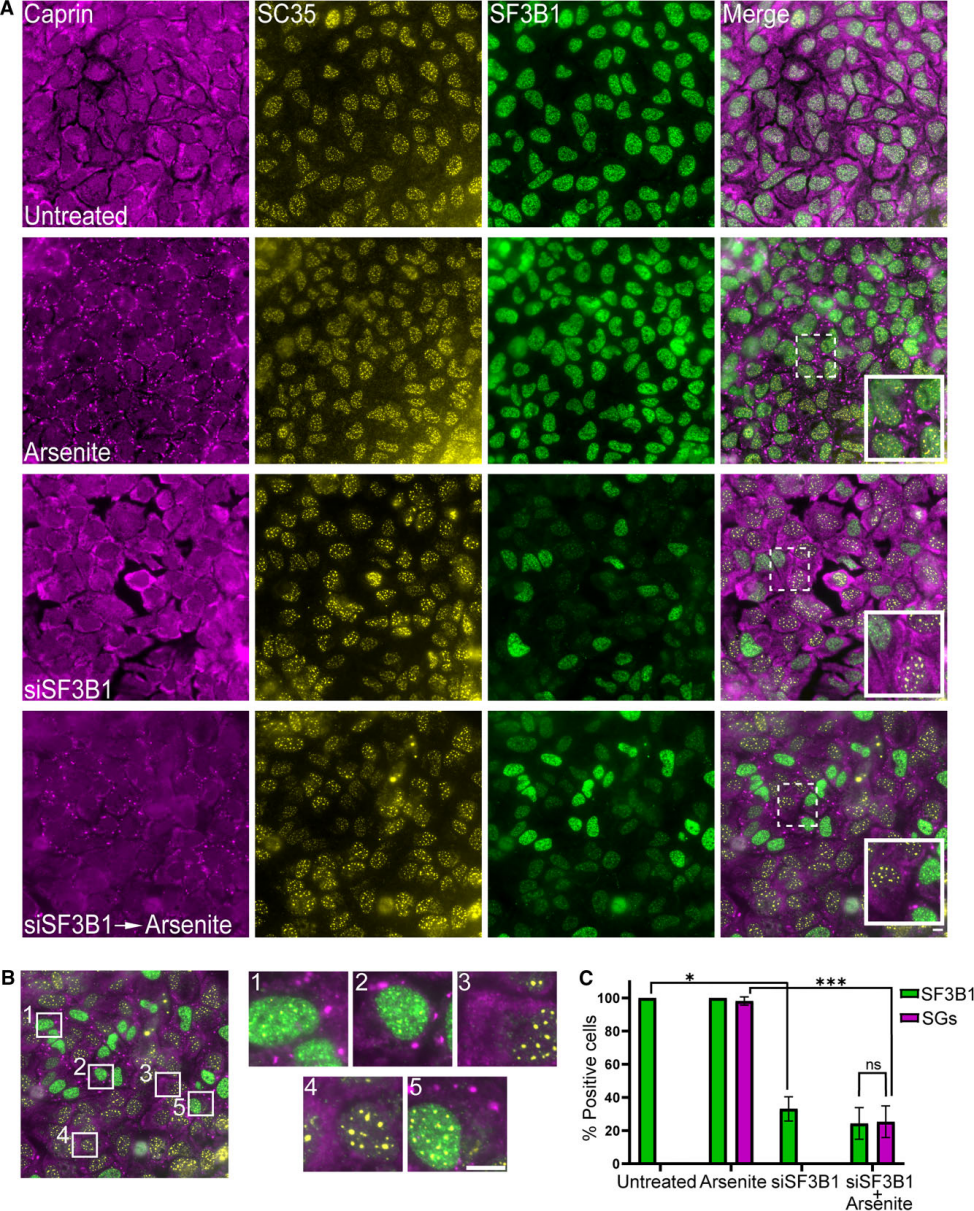
SF3B1 knockdown suppresses SG formation. (**A**) U2OS cells were treated with siRNA targeting SF3B1 for 72 h. Arsenite (0.25 mM) was added 30 min before fixation. Cells were stained with anti-Caprin as an SG marker (magenta), anti-SC35 as a nuclear speckle marker (yellow) and anti-SF3B1 (green). Scale bars = 10 μm. Enlargements of designated areas are in the boxed regions.(**B**) (Left) The merged image of the cells treated with siRNA targeting SF3B1 followed by arsenite from panel (A) (bottom right image) showing (right) enlargements of the merged channels of certain cells from the field, as numbered. (**C**) Quantification of the population of SG-positive and SF3B1-positive U2OS cells treated as described in panel (B). Cells were counted in three independent experiments (*n* > 400 cells per marker). Data were analyzed using one-way ANOVA for groups without mean of 0 or 100, followed by Tukey’s post hoc analysis. For comparison of groups with mean 0 or 100, one-sample *t*-tests against 0 and 100 were performed (*P< 0.05, ***P< 0.001, ns = nonsignificant). Bar graph illustrates the mean ± standard deviation.

It is well known that transcription and splicing are tightly linked (37). In continuation with the above findings that transcription inhibition has a negative effect on SG formation, and in light of the lack of studies examining the effect that splicing inhibition might have on SGs, we examined whether splicing inhibition affects SG formation. To address this issue, we applied the commonly used splicing inhibitor PLB, which inhibits SF3B1, an important component in complex A of the spliceosome (38). Treatment with PLB for 6 h led to an enlargement in the size of nuclear speckles (39), a known indication for inhibition of pre-mRNA splicing. In addition, this treatment also resulted in reduced size of arsenite-induced SGs, compared to cells treated with arsenite only (Figure 2A and B). Strikingly, applying PLB for longer times (24 h) led to an almost complete inhibition of SG formation in arsenite-treated cells (∼3% cells positive for SGs). Under these conditions, TIA1 localized mostly in the nucleus (Figure 2C and D), compared to ActD treatment that suppressed SG formation as well, in which TIA1 was observed dominantly in the cytoplasm (Figure 1A). This effect on SG formation was not limited to arsenite only, as an inducer of SG formation, but was also observed under various stress types such as thapsigargin, which leads to endoplasmic reticulum (ER) stress (40), and VRB, a chemotherapy that targets the microtubules (41) (Supplementary Figure S2). As mentioned above, PLB targets SF3B1. Therefore, we wanted to verify that the direct inhibition of SF3B1 leads to the blockage of SG formation, and rule out the possibility that the observed impact of PLB on SGs is due to off-target effects. To address this issue, we knocked down SF3B1 levels using siRNA, and then treated the cells with arsenite. Indeed, cells that did not contain the SF3B1 protein showed a significant decrease in SG formation when arsenite treatment was applied (Figure 3). These results suggest that interfering with the splicing process by targeting SF3B1 has a negative effect on SG assembly.

### Splicing inhibition negatively affects SG formation

**Figure 4.**
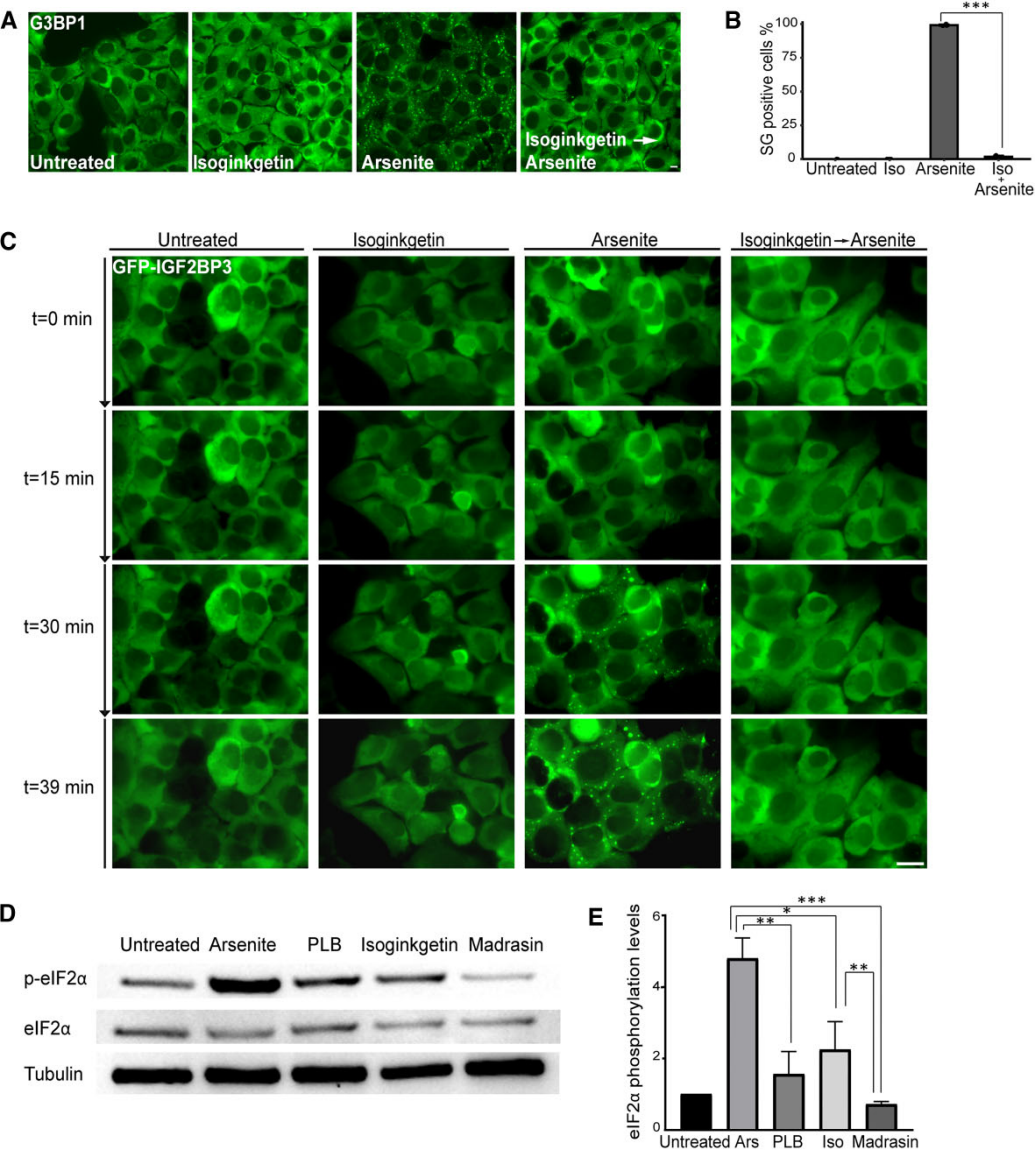
Splicing inhibition by isoginkgetin prevents the formation of arsenite-induced SGs. (**A**) U2OS cells were treated with isoginkgetin (Iso, 100 μM; 4 h) and arsenite (0.25 mM) was added 30 min before fixation. Cells were stained with anti-G3BP1 as an SG marker. Scale bar = 10 μm. (**B**) Quantification of the population of SG-positive U2OS cells treated as described in panel (A). Cells were counted in three independent experiments (*n* > 180 cells per treatment). Data were analyzed with independent sample *t*-tests (***P< 0.001). Bar graph illustrates the mean ± standard deviation. (**C**) Frames from live-cell movies of GFP-IGF2B3 stably expressing cells under the following conditions: untreated, treated with arsenite, treated with isoginkgetin (100 μM; 4 h) and followed by the addition of arsenite (0.25 mM) at the starting time point (*t* = 0 min). Scale bar = 10 μm. (**D**) Western blot of protein extracts from U2OS cells treated with arsenite (Ars, 0.25 mM, 1 h), isoginkgetin (100 μM, 4 h), PLB (0.5 μM, 6 and 24 h) and madrasin (30 μM, 4 h). Blots were incubated with anti-eIF2α, anti-p-eIF2α and anti-tubulin for loading control. (**E**) Quantification of the eIF2α phosphorylation levels from panel (D). Phosphorylation levels in all samples were normalized to the total eIF2α protein level in each sample, and all samples were normalized to the untreated signal, and then the data were log transformed. Data were analyzed using ImageJ, and statistical analysis was carried out by one-way ANOVA followed by Tukey’s post hoc analysis (*n* = 3, *P< 0.05, **P< 0.01, ***P< 0.001). Bar graph illustrates the mean ± standard deviation.

Since targeting SF3B1 had a negative effect on SG formation, we explored whether this effect was generally due to splicing inhibition. To this end, we used the splicing inhibitor isoginkgetin that prevents the assembly of complex B of the spliceosome but does not target SF3B1 (42). Indeed, pretreating the cells with isoginkgetin (100 μM for 4 h) led to an almost complete inhibition of the formation of arsenite-induced SGs. The population of cells that were positive for SGs declined from ∼99% in arsenite treatment only to ∼1.5% in cells that were pretreated with isoginkgetin (Figure 4A and B). This was followed in living cells; pre-incubating the cells for 4 h with isoginkgetin prevented arsenite-induced SG formation compared to arsenite alone (Figure 4C and Supplementary Movies 1–4). The effect of isoginkgetin on arsenite-induced SGs was also demonstrated on A549 human lung adenocarcinoma epithelial cells (Supplementary Figure S3A). This suggests that the effect of splicing inhibition negatively affects the assembly of SGs.

SGs form following the phosphorylation of eIF2α (43). Therefore, we examined whether the splicing inhibitors affect eIF2α phosphorylation. Cells were treated with PLB, isoginkgetin and an additional splicing inhibitor, madrasin (44). Surprisingly, we found that the splicing inhibitors differently affected eIF2α phosphorylation. Isoginkgetin led to a slight increase in eIF2α phosphorylation, while madrasin led to the opposite effect (Figure 4D and E). Furthermore, we tested whether isoginkgetin leads to changes in eIF2α phosphorylation levels under arsenite treatment. There was no difference in the phosphorylation levels between arsenite treatment alone or combined with isoginkgetin (Supplementary Figure S3B and C). These results, together with the suppression of SG assembly while splicing or transcription was inhibited, strengthen the assumption that splicing inhibition affects SG formation due to the lack of mRNA in the cytoplasm, which affects the ability to initiate the formation of SGs in the cytoplasm, and not by directly affecting the ISR pathway.

### Madrasin induces the formation of stress-like granules

**Figure 5.**
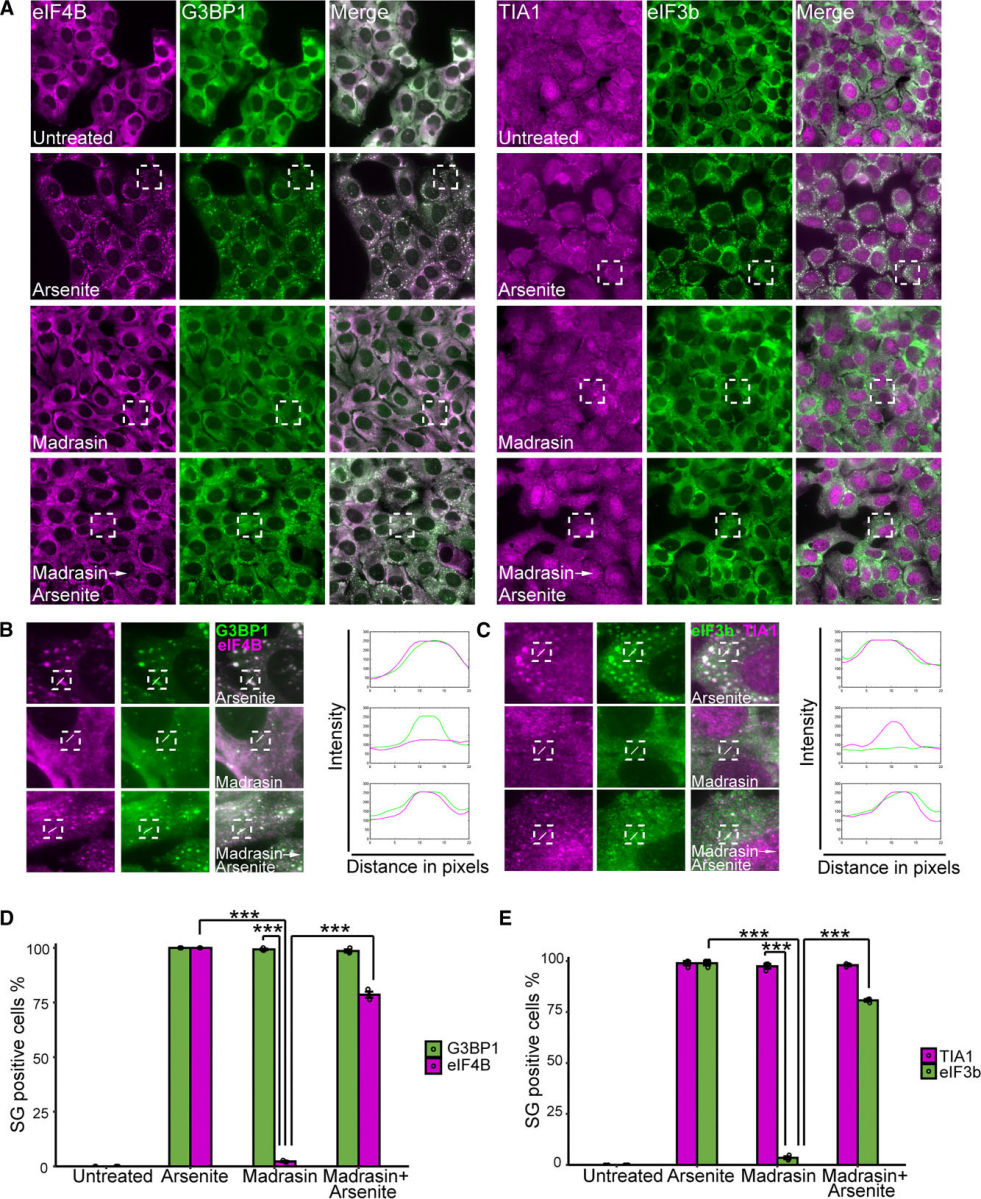
Madrasin induces the formation RNA-containing stress-like granules. (**A**) U2OS cells were treated with madrasin (30 μM; 4 h) and with arsenite (0.25 mM; 30 min) before fixation. Cells were stained with anti-eIF4B (magenta, left panel) and anti-G3BP1 (green, left panel), or with anti-TIA1 (magenta, right panel) and anti-eIF3b (green, right panel). (**B**) (Left) Enlargements of designated areas in the boxed regions in panel (A), marked by anti-eIF4B (magenta) and anti-G3BP1 (green). (Right) Intensity analysis from the boxed region along the white line. (**C**) (Left) Enlargements of designated areas in the boxed regions in panel (A), marked by anti-TIA1 (magenta) and anti-eIF3b (green). (Right) Intensity analysis from the boxed region along the white line. Quantification of the population of SG-positive U2OS cells, (**D**) marked by G3BP1 and eIF4B or (**E**) marked by TIA1 and eIF3b, treated as described in panel (A). Cells were counted in three independent experiments (*n* > 180 cells per treatment and marker). Data were analyzed with one-way ANOVA, followed by Tukey’s post hoc analysis (***P< 0.001). Bar graph illustrates the mean ± standard deviation.

Following the findings that the splicing inhibitors differently affect eIF2α phosphorylation, we were interested in examining whether madrasin treatment would inhibit arsenite-induced SG assembly, as observed for the other splicing inhibitors. Surprisingly, staining for hallmark SG markers TIA1 and G3BP1, on madrasin-only treated cells, revealed that madrasin treatment led to the formation of small cytoplasmic granules that contain mRNAs, in U2OS and A549 cells (Supplementary Figures S3D and S4A and B). Since the translation initiation complex proteins eIF3b and eIF4B that are also regulated by mTOR (45) are typically found in SGs, we examined whether they localized in the madrasin-induced granules. The granules did not contain these hallmark SG proteins, and so we termed them stress-like granules. However, treating the cells with madrasin followed by arsenite treatment led to the appearance of eIF4B and eIF3b within the granules in ∼75% of the cells (Figure 5). These findings could suggest that madrasin leads to the initiation of an intermediate granule that cannot fully mature, and that the subsequent arsenite treatment drives the relocalization of these proteins to a more mature SG stage.

SGs form as a result of translation arrest and ribosome runoff (20). Staining for the small ribosomal subunit protein 6 (RPS6), which is a common SG marker and indicates ribosome runoff (46), showed that RPS6 is not localized in the madrasin-induced granules. Here, the addition of arsenite did not lead to the appearance of RPS6 in the granules, enhancing the possibility that the cytoplasmic stress-like granules formed by madrasin are not the classic SGs (Supplementary Figure S4C–E). Moreover, treating stressed cells with the translation inhibitor cycloheximide, a polysome stabilizer, typically brings to the disassembly of SGs (20). Since the madrasin-induced granules were not canonical SGs, it was interesting to determine whether the assembly of these granules will be blocked by cycloheximide. Treating the cells with cycloheximide after madrasin treatment had no significant effect on stress-like granule formation, suggesting that madrasin-induced granules are not an intermediate step in SG assembly (Supplementary Figure S5A and B). VRB, a chemotherapy that induces canonical SGs (23,41), served as a positive control for the cycloheximide treatment (45).

Although we previously found that madrasin treatment did not elevate eIF2α phosphorylation levels (Figure 4D and E), it did induce the formation of stress-like granules. We next examined whether their formation would be inhibited by ISRIB, a small molecule that blocks the effect of eIF2α phosphorylation. eIF2α is part of the ternary complex required for translation initiation, which is inhibited by its phosphorylation under various stress conditions, leading to SG formation (47). Treating the cells with ISRIB prior to arsenite alone led to an almost complete blockage of canonical SG formation, as expected (Supplementary Figure S5C and D). However, the granules formed by madrasin were not affected by ISRIB pretreatment. Using western blotting, we verified that ISRIB indeed led to a decrease in eIF2α phosphorylation levels under these conditions (Supplementary Figure S5E and F). Altogether, these results suggest that madrasin leads to the assembly of G3BP1- and TIA1-associated mRNA cytoplasmic stress-like granules, but not to the formation of SGs *per se*, formed via the p-eIF2α mechanism.

Finally, since madrasin and isoginkgetin had a dramatic effect on SG formation, we wanted to determine whether these were reversible effects, and whether SGs can re-form after washing out of the inhibitors and treating with arsenite. Indeed, washing the cells for 24 h after treatment with isoginkgetin or madrasin restored splicing (Supplementary Figure S6), and SGs induced by arsenite were able to form again (Supplementary Figure S7). Moreover, after washing the cells for 24 h to restore splicing, madrasin-induced stress-like granules disappeared, and the cells were able to form large SGs induced by arsenite (Supplementary Figure S7A and C). To rule out the possibility that SG formation is inhibited by transcription inhibition that may occur during splicing inhibition, staining of nascent RNA synthesis was performed using EU (Supplementary Figure S8A) and a quantification of active sites of transcription in splicing-inhibited cells was performed (Supplementary Figure S8B and C). These results demonstrated that the splicing inhibitors did not have a similar effect on transcription as did the transcription inhibitor ActD at high concentrations used to block RNAPII.

### Splicing inhibitors affect other RNA granules

**Figure 6.**
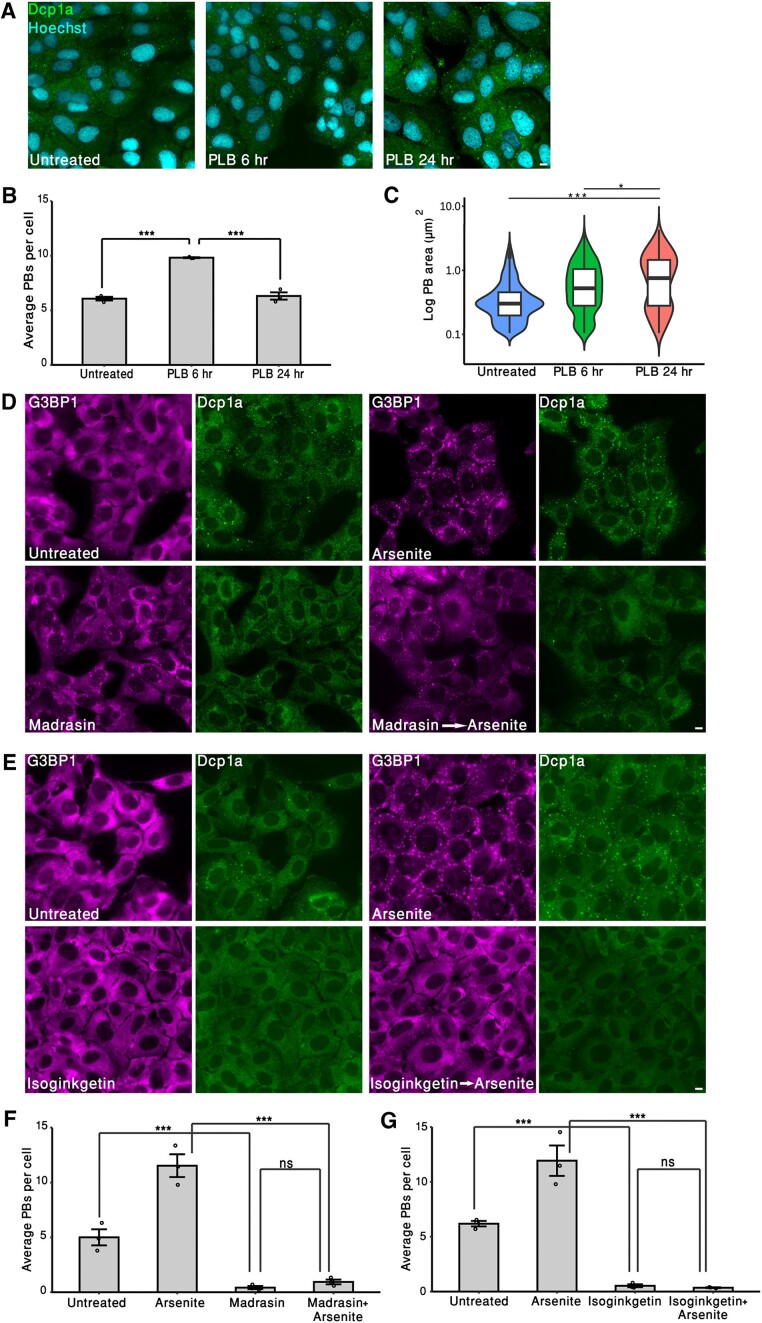
The effect of splicing inhibitors on PBs. (**A**) U2OS cells were treated with PLB (0.5 μM) for 6 or 24 h, and cells were stained with anti-Dcp1a to mark PBs (green). DNA Hoechst staining is in cyan. Scale bars = 10 μm. (**B**) Quantifications representing average PB numbers per cell. U2OS cells were treated as described in panel (A), and then data were log transformed (base 2) to meet normality assumption. Data were analyzed using ANOVA, followed by Tukey’s post hoc analysis (*n* = 3, >220 cells were quantified per treatment). Bar graph illustrates mean ± standard deviation. (**C**) Violin plot representing PB area in U2OS cells treated as described in panel (A). Data were analyzed using one-way nested ANOVA. More than 330 PBs were quantified for each experiment per treatment (*n* = 3). The graph represents one experiment, and the statistical test for significance was carried for all three independent experiments (***P< 0.001, *P< 0.05). (**D**) U2OS cells were treated with madrasin (30 μM; 4 h). Arsenite (0.25 mM) was added 30 min before fixation, and cells were stained with anti-Dcp1a to mark PBs (green) and anti-G3BP1 to mark SGs (magenta). (**E**) U2OS cells were treated with isoginkgetin (100 μM; 4 h). Arsenite (0.25 mM) was added 30 min before fixation, and cells were stained with anti-Dcp1a to mark PBs (green) and anti-G3BP1 to mark SGs (magenta). (**F**) Quantifications representing average PB numbers per cell. U2OS cells were treated as described in panel (D). Data were analyzed as described in panel (B), and >200 cells were quantified per treatment (*n* = 3, ***P< 0.001, ns = nonsignificant). (**G**) Quantifications representing average PB numbers per cell. U2OS cells were treated as described in panel (E). Data were analyzed as described in panel (B), and >290 cells were quantified per treatment (*n* = 3, ***P< 0.001, ns = nonsignificant).

RNA granules are a large family of protein assemblies containing RNAs, including SGs, PBs, nuclear speckles, neuronal granules and more (4,9). Looking at the effects that splicing inhibitors had on SGs led us to examine whether splicing inhibitors would affect PBs. These are cytoplasmic granules, associated with RNA decay and storage, that are always present in the cytoplasm (48,49). Cells were treated with PLB (6 or 24 h) and stained for Dcp1a, a hallmark protein marker for PBs. PLB treatment for 6 h led to a significant rise in PB abundance, while applying PLB for 24 h did not increase PB numbers, but increased PB size significantly (Figure 6A–C).

We then examined whether the other splicing inhibitors affect PBs. Surprisingly, madrasin and isoginkgetin treatment led to almost a complete dissolution of PBs (Figure 6D–G). It is known that arsenite treatment induces SGs as well as PB formation (50). Therefore, we examined PB formation under madrasin or isoginkgetin, followed by arsenite treatment. Arsenite treatment alone promoted a rise in PB numbers, as expected. However, adding arsenite to cells that were pretreated with madrasin or isoginkgetin did not rescue PB formation (Figure 6D–G). These observations suggest that, first, inhibition of splicing can affect the formation of various RNA granules, and not only SGs, and, second, inhibiting splicing, specifically by madrasin or isoginkgetin treatment, suppresses the formation of different cytoplasmic RNA granules under stress conditions.

It is known that splicing inhibition leads to an enlargement of nuclear speckles (51). The observation that different splicing inhibitors affect PBs differently led us to question whether nuclear speckles would be enlarged in cells treated with the other splicing inhibitors. Indeed, we noticed that PLB treatment caused a significant enlargement of nuclear speckles, yet isoginkgetin treatment reduced the structure of the nuclear speckles (Supplementary Figure S9A and B). The effect of splicing inhibitors on nuclear speckles and PBs was also demonstrated in A549 cells (Supplementary Figure S9C). Altogether, these results reveal that splicing inhibitors not only affect SGs, but also have an impact on other granules, nuclear or cytoplasmic.

### Blocking mRNA export prevents SG formation

Our findings suggest that splicing inhibitors suppress SG formation independently of eIF2α phosphorylation. Together with the knowledge that mRNAs are essential for SG formation, are important for PB assembly and are spliced and exported from the nucleus to the cytoplasm (52), we postulated that the effect of splicing inhibition on SG formation might lie in the shortage of mRNA in the cytoplasm. Therefore, we asked whether the prevention of mRNA export to the cytoplasm will impede SG formation. We first verified that the localization of the mRNA population in the cell subcompartments is affected while splicing is inhibited. Cells were treated with PLB for 6 h, or with isoginkgetin for 4 h, and RNA FISH that detects poly(A)^+^ transcripts was performed. Typically, poly(A)^+^signal is seen in the cytoplasm and in nuclear speckles in the nucleus. Here, we found that the poly(A)^+^ signal increased in the nucleus after splicing inhibition, a known indicator of export inhibition (25). Washing the cells for 24 h after treating with splicing inhibitors restored the export of these transcripts from the nucleus to the cytoplasm (Supplementary Figure S10A). We further verified that splicing inhibition blocks mRNA export by examining the subcellular localization of the abundant mRNA transcript MKI (29). Using single-molecule mRNA FISH, we observed that under PLB treatment MKI transcripts were found predominantly in the nucleus (Supplementary Figure S10B). Quantification of MKI transcripts in the cytoplasm showed that their numbers decreased significantly in the splicing-inhibited cells (Supplementary Figure S10C). Since ribosome runoff promotes SG formation, and the stabilization of polysomes suppresses SG formation (20), we questioned whether the splicing inhibition has an effect on the association of mRNA with polysomes. Therefore, we performed polysome profiling analysis, which revealed a reduction of RNA in polysomes under PLB treatment (Supplementary Figure S10D), indicative of the shortage of mRNA in the cytoplasm under these conditions. Altogether, the above findings imply that a lack of mRNAs while splicing is inhibited might play an important role during the state in which stressed cells do not form SGs. This suggests that splicing inhibition leads to an mRNA export block, which depletes the amount of cytoplasmic mRNA that is available and necessary for SG formation.

**Figure 7.**
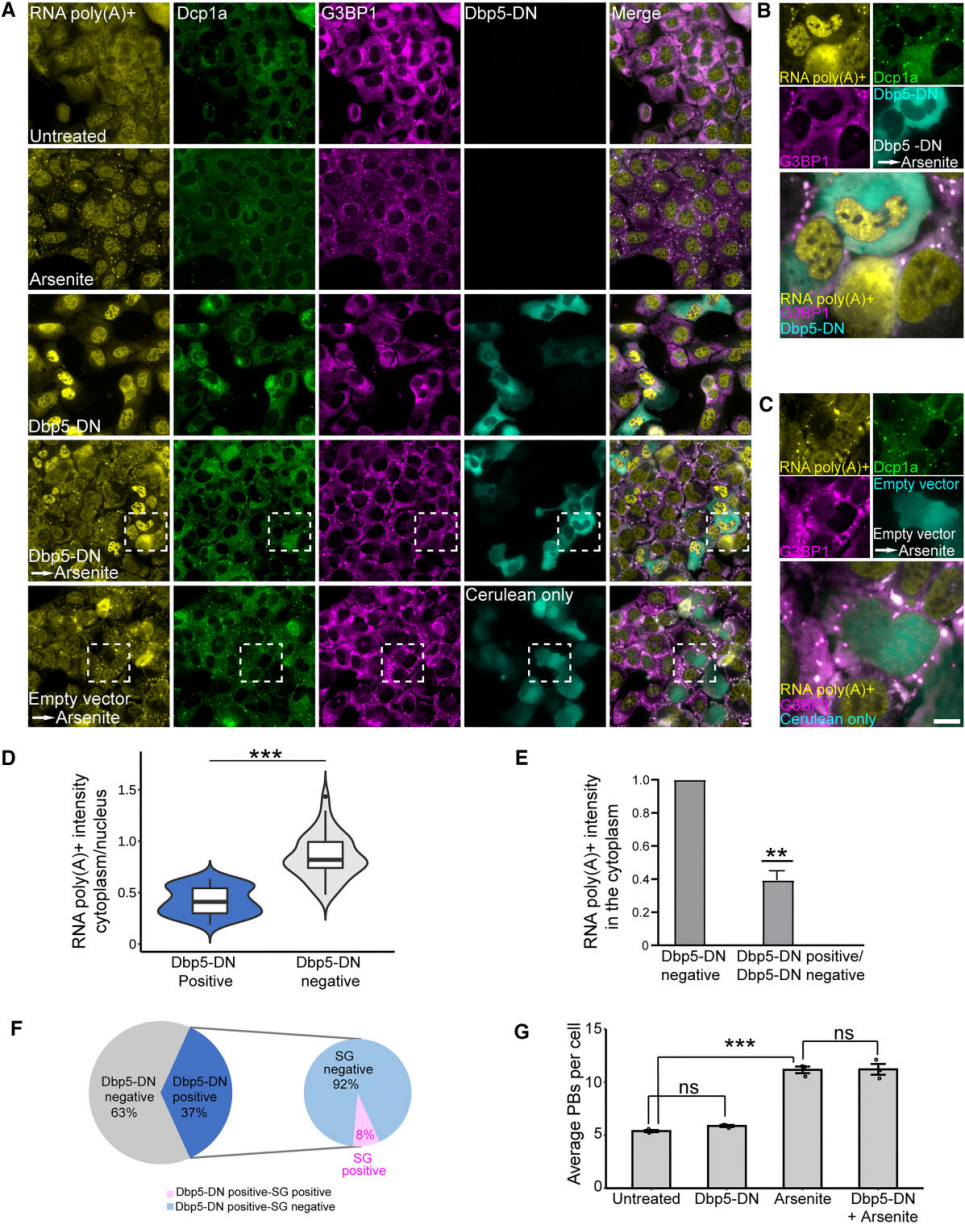
The effect of export inhibition on cytoplasmic RNA granules. (**A**) U2OS cells were transfected for 24 h with Cerulean-Dbp5-DN (cyan) to block mRNA export or with an empty Cerulean plasmid as a control, using Lipofectamine 2000. Arsenite (0.25 mM) was added 30 min prior to fixation, and cells were stained by RNA FISH using a fluorescent probe to poly(A)^+^ RNA (yellow), followed by immunofluorescence with anti-Dcp1a as a marker for PBs (green) and anti-G3BP1 as a marker for SGs (magenta). Scale bar = 10 μm. Enlargements of the marked boxes in panel (A): (**B**) the transfected Dbp5-DN cells and (**C**) the Cerulean control plasmid, under arsenite as described in panel (A). (**D**) Violin plot representing the ratio of the intensity of the RNA poly(A)^+^ signal in the cytoplasm and nucleus. More than 80 cells were quantified (*n* = 3, ***P< 0.001). Data were analyzed using independent sample *t*-tests. (**E**) Quantification of poly(A)^+^ RNA intensity in the cytoplasm of U2OS cells that were transfected with Cerulean-Dbp5-DN (24 h) to block mRNA export and treated with arsenite (30 min, 0.25 mM). Intensity in the cytoplasm of the transfected cells was measured by ImageJ and was normalized to the intensity of the cytoplasm of cells that were negative for the Cerulean-Dbp5-DN transfection. Data were log transformed and statistical analysis was performed by one-sample *t*-test (against a constant mean of 0; **P< 0.01, >50 cells were quantified, *n* = 3). (**F**) Pie chart representing the percentage of Dbp5-DN-positive (blue) and Dbp5-DN-negative (gray) cells. The right pie chart represents the percentage of SG-positive cells (pink) and SG-negative cells (cyan), in the Dbp5-DN-positive population. More than 100 cells were quantified per each condition (*n* = 3). (**G**) Quantification of average number of PBs per cell in U2OS cells treated as described in panel (A). More than 90 cells were quantified (*n* = 3), and data were analyzed with one-way ANOVA, followed by Tukey’s post hoc analysis (***P< 0.001, ns = nonsignificant). Bar graph illustrates the mean ± standard deviation.

To validate that the prevention of SG formation in stressed cells is indeed due to the prevention of mRNA export while splicing is inhibited, we directly blocked nuclear export using the Dbp5-DN helicase (25). Dbp5 helicase is essential for mRNA export and therefore high expression of Dbp5-DN prevents the export of mRNA to the cytoplasm. We inhibited mRNA export by transfecting Dbp5-DN to the cells, and then applied arsenite to observe whether SGs formed. Indeed, arsenite-treated cells that showed high expression of Dbp5-DN did not form SGs (Figure 7A–D). Specifically, ∼92% of the cell population that was Dbp5-DN-positive lacked SGs, and mRNA levels in the cytoplasm were significantly reduced in the export-inhibited cells (Figure 7E and F). However, export inhibition did not affect PB numbers (Figure 7G). To verify that the observed effect is due to export inhibition, we blocked mRNA export by a different mechanism. By increasing the levels of FG repeats (of nucleoporins) in the nucleoplasm, we could block mRNA export, as previously shown (25,53). Here too, export-blocked cells did not show SGs (Supplementary Figure S11). Considering these results, we suggest that export inhibition, which can also be achieved by transcription or splicing inhibition, leads to a depletion of mRNAs in the cytoplasm, and therefore has a negative effect on SG formation induced by arsenite, yet does not affect PB formation.

### Transfection of synthetic mRNA directly into the cytoplasm leads to SG formation

It was suggested by Child *et al.* (21) that transcripts that are newly exported from the nucleus to the cytoplasm obtain a nucleating property that promotes the assembly of SGs. To answer the question whether nascent transcripts are essential for SG formation, nascent RNAs were labeled by an EU pulse, and cells were then treated with PLB and arsenite. Nascent mRNAs were observed in arsenite-induced SGs, but not in the SGs that were assembled in cells that were pretreated with the splicing inhibitor PLB for 6 h (Supplementary Figure S12). This suggests that the presence of nascent mRNAs in the cytoplasm might not be the only limiting factor for SG formation under the conditions of splicing inhibition, but rather the global amount of mRNA transcripts in the cytoplasm.

**Figure 8.**
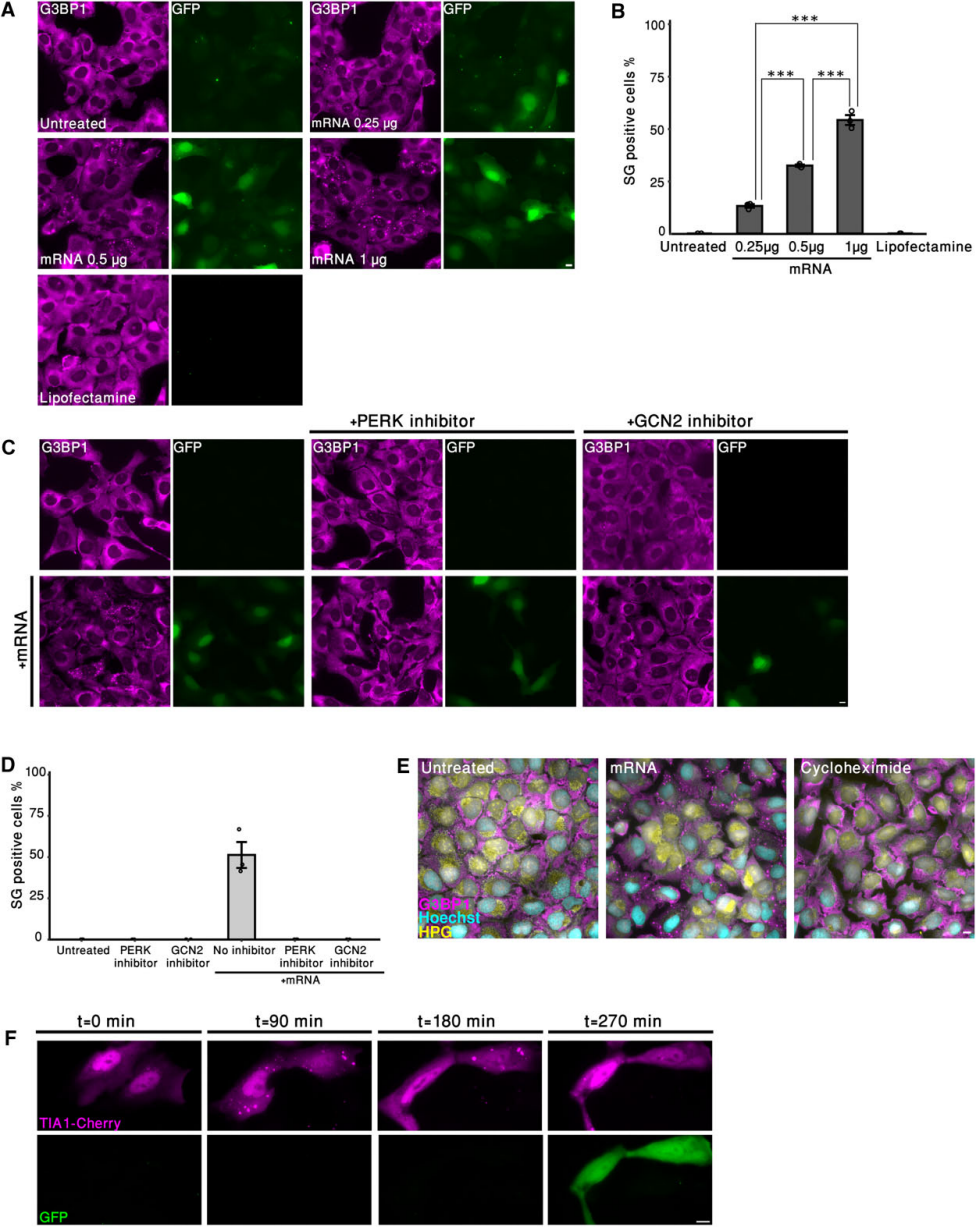
Delivery of mRNA into the cytoplasm induces SG formation. (**A**) U2OS cells were transfected with *in vitro* transcribed mRNA (0.25, 0.5 or 1 μg) encoding the protein GFP (green) using Lipofectamine 2000, and fixed after 1.5 h. Cells were stained using anti-G3BP1 (magenta). Scale bars = 10 μm. (**B**) Quantification of the population of SG-positive U2OS cells treated as described in panel (A). Cells were counted in three independent experiments (*n* > 245 cells per treatment). Data were analyzed with one-way ANOVA, followed by Tukey’s post hoc analysis (***P< 0.001). Bar graph illustrates the mean ± standard deviation. (**C**) U2OS cells were treated with a PERK inhibitor (GSK II, 40 μM), or with a GCN2 inhibitor (4 μM), for 3 h prior to transfection. mRNA (1 μg) encoding GFP (green) was transfected, and cells were fixed 1.5 h post-transfection. Cells were stained using anti-G3BP1 (magenta). (**D**) Quantification of the cell population positive for SGs in the conditions as described in panel (A). More than 190 cells were quantified (*n* = 3). Statistical significance was tested using one-sample *t*-test (*P< 0.05). Bar graph illustrates the mean ± standard deviation. (**E**) U2OS cells were transfected with mRNA (1 μg) encoding GFP or treated with cycloheximide (100 μg/ml) for 2 h. Cells were stained using Click-iT HPG (yellow) to detect cells with active translation and anti-G3BP1 (magenta). Elongated cytoplasmic structures (yellow) as in the left panel are indicative of active translation. Nascent protein synthesis is assessed by determining signal intensity in the fluorescent channel in the ring around the nucleus as defined by Hoechst DNA stain in cyan. (**F**) Frames from a live-cell movie of mCherry-TIA1 expressing cells, transfected with mRNA (1 μg) encoding GFP (green).

New transcripts that are exported from the nucleus to the cytoplasm are coated with a variety of RNBPs such as export, splicing and shuttling proteins, and some of these factors such as nuclear RNA export factor 1 (NXF1) can colocalize in SGs (27). Therefore, we wanted to understand whether a lack of these endogenous RNBPs on the transcript would limit SG formation. To answer this question, mRNA encoding GFP was prepared *in vitro*, and this exogenic mRNA was transfected into the cytoplasm directly, thereby bypassing the nucleus and the endogenous pathway of mRNP formation by binding of RNBPs. Interestingly, the transfection of the *in vitro* transcribed mRNA alone led to the formation of SGs without applying additional stress. Furthermore, increasing the quantity of transfected mRNA increased the number of cells positive for SGs. Transfecting mRNA at a concentration of 0.25 μg mRNA resulted in ∼13% of cells that were positive for SGs, while transfecting 0.5 and 1 μg of mRNA raised the SG-positive population to ∼33% and ∼54%, respectively. Lipofectamine, the transfection reagent, had no effect on SG formation (Figure 8A and B). We also verified that that the SGs formed while transfecting different types of mRNAs, and ensured that a shorter mRNA synthesized from the same plasmid did not induce SG formation as the full-length transcript (Supplementary Figure S13). These results strengthen the idea that the amount of mRNA transcripts in the cytoplasm has an important impact on the assembly of SGs.

Next, we were interested in elucidating the mechanism by which these granules formed. We first used ISRIB that inhibits SG assembly following the phosphorylation of eIF2α (47). Incubating the cells with ISRIB prior to the transfection significantly reduced SG formation caused by the transfected mRNA (Supplementary Figure S14A and B). This indicated that the SGs induced by the transfected mRNA assembled as a result of eIF2α inhibition, which is achieved by eIF2α phosphorylation. We next determined which kinase of the ISR was responsible for the phosphorylation eIF2α under the mRNA transfection conditions. The cells were incubated with PKR, GCN2 or PERK inhibitors, and then the *in vitro* transcribed mRNA was transfected. We found that inhibiting PKR did not affect SG formation, yet PERK or GCN2 inhibition blocked the assembly of SGs induced by mRNA transfection (Figure 8C and D, and Supplementary Figure S14C). These results indicate that although a large quantity of mRNA transcripts in the cytoplasm can lead to SG formation, kinases that are related to protein synthesis stress (i.e. PERK and GCN2) are activated, directly or indirectly, by the presence of these mRNAs.

Since we found that kinases that are related to ER stress and amino acid deprivation are activated due to mRNA transfection, we examined whether these SGs formed directly by the abundance transcripts in the cytoplasm or by ER stress caused by massive protein synthesis. Namely, we asked whether ISR kinases are activated prior to protein translation and while SGs are observed. Therefore, we examined the presence of nascent protein synthesis in the transfected cells using l-homopropargylglycine (HPG), an analogue of methionine. In this technique, the cells are incubated with methionine-free medium after treatment, and then HPG is added, which after fixation is conjugated to a fluorophore. Finally, nascent protein synthesis is observed by the intensity of the fluorophore around the nucleus, using microscopy. Although we found that these SGs formed via the activation of PERK or GCN2, cells that were positive for SGs showed little nascent protein synthesis, suggesting that the SGs appearing after mRNA transfection were formed directly by the mRNAs and prior to protein synthesis (Figure 8E). To verify that the SGs formed prior to protein synthesis, we performed live-cell imaging. Indeed, SGs were observed ∼90 min after mRNA transfection, and at this point the GFP protein was not observed yet. After ∼270 min, SGs disassembled and protein translation occurred (Figure 8F and Supplementary Movies 5 and 6). Altogether, we suggest that an abundance of cytoplasmic mRNA leads to the activation of protein synthesis stress kinases (i.e. GCN2 and PERK), which in turn leads to SG formation, until eventually stress is resolved, and protein translation occurs.

Finally, we wanted to determine whether the hallmark SG proteins G3BP1/2 are essential for the formation of the granules formed due to the mRNA transfection. It was demonstrated previously using ΔΔG3BP1/2 U2OS knockout cells (8) that these proteins are essential for SG formation under various stress types. For example, it was shown that these cells do not form SGs under the conditions of chemotherapy and chronic starvation (7,41). Indeed, ΔΔG3BP1/2 cells did not form SGs after transfecting *in vitro* transcribed mRNA, in comparison to regular U2OS cells (Supplementary Figure S14D). This indicates that G3BPs are essential for the formation of these SGs, even though these granules form directly from an abundance of mRNAs in the cytoplasm, emphasizing the importance of RNA–protein interactions in the assembly of SGs.

### mRNA transfection partially rescues SG formation while splicing is inhibited

**Figure 9.**
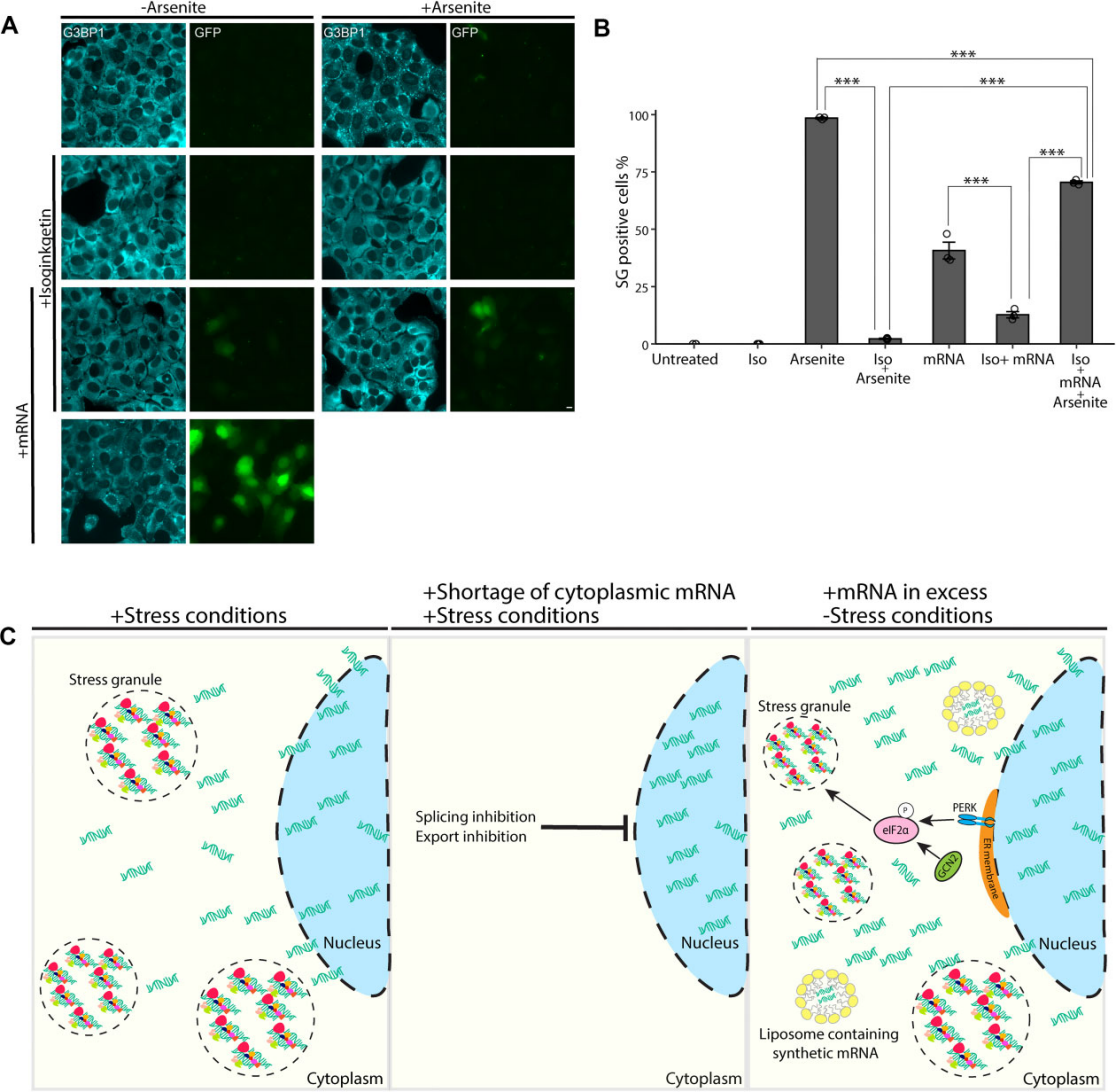
mRNA transfection partially rescues SG formation under splicing inhibition followed by arsenite treatment. (**A**) U2OS cells were treated with isoginkgetin (Iso, 50 μM) for 5 h overall, transfected with mRNA (0.75–1 μg) encoding GFP (green) for 1.5 h and treated with arsenite 30 min before fixation. Cells were stained with anti-G3BP1 to mark SGs (cyan). Scale bar = 10 μm. (**B**) Quantification of the population of SG-positive U2OS cells treated as described in panel (A). Cells were counted in three independent experiments (*n* > 260 cells per treatment). Data were analyzed with independent sample *t*-tests or with one-way ANOVA, followed by Tukey’s post hoc analysis (***P< 0.001). Bar graph illustrates the mean ± standard deviation. (**C**) Illustration of the suggested model. (Left) Under stress conditions, SGs are formed. (Middle) Shortage of mRNA in the cytoplasm due to the inhibition of splicing or export prevents the formation of SGs under stress conditions. (Right) Excess of cytoplasmic mRNA leads to SG formation, via the activation of PERK and GCN2, without applying any additional stress.

Since splicing inhibition and mRNA export blockage led to the suppression of SG formation, we speculated that the limiting factor for SG formation was the shortage of mRNAs in the cytoplasm. Therefore, we expected that adding synthetic mRNAs to cells in which splicing was inhibited might rescue SG formation. To test this, cells were first treated with the splicing inhibitor isoginkgetin, which completely blocked SG assembly in arsenite-treated cells (Figure 4). Then, *in vitro* transcribed mRNA encoding for GFP was transfected, and cells were treated with arsenite. Indeed, the transfected mRNA rescued SG formation under the latter conditions (Figure 9A). A negligible population of cells was positive for SGs under the treatment of isoginkgetin (50 μM) followed by arsenite treatment (∼2.5%), while adding the mRNA raised the number of positive cells significantly (∼70%). It is important to mention that cells that were treated with isoginkgetin and then underwent transfection showed SGs, but significantly less than the ones that were treated with arsenite as well under these conditions (∼13% versus ∼70%; Figure 9B). In addition, although the transfection of mRNA increased the percentage of cells that were positive for SGs, it was well observed that unlike arsenite treatment alone, not all cells were positive for SGs (∼98.5% versus ∼70%). Altogether, we suggest that mRNA quantity in the cytoplasm is one of the mechanisms that regulates SG formation; a shortage of mRNAs in the cytoplasm, due to splicing, transcription or export inhibition, suppresses SG formation (Figure 9C), and this can be partially rescued by excess mRNAs in the cytoplasm.

## Discussion

RNA has an important role in the assembly and the determination of SG physical properties (18,54–56). In general, the formation of SGs relies on the presence of nonpolysomal mRNAs in the cytoplasm (57). In this respect, cycloheximide, which prevents polysome dissociation, inhibits SG formation, while puromycin, which dissociates polysomes, promotes SG formation, but is not sufficient for driving massive SG assembly (20,58). Here, we show that inhibition of nascent RNA synthesis, maturation or export leads to a suppression in SG formation, reducing SG size and quantity. We suggest that the importance of newly transcribed mRNA in SG assembly lies mostly in the quantity of the untranslated mRNAs.

Previous studies have shown that transcription inhibition by ActD blocks SG formation (20,59,60). It was suggested that changes in the subcellular localization of shuttling nuclear RNBPs such as TIA1 and HuR from the nucleus to the cytoplasm might stabilize the mRNA and this could promote the dissociation of SGs or would prevent their formation altogether; i.e. excess of TIA1 localization in the cytoplasm could have an inhibitory effect on SG formation (20). We find that pretreating cells with various transcription inhibitors, including ActD, which mainly affects transcription associated with RNAPII, suppressed SG formation. Then, we showed that under splicing inhibition by PLB, SG formation was prevented under various stressors, whereas the shutting RNBP TIA1 was found predominantly in the nucleus (Figures 1 and 2). This implies that the presence of TIA1 in the cytoplasm is not always a prerequisite for preventing SG formation. Therefore, we speculated that a shortage of newly exported mRNA to the cytoplasm might be a reason for the impairment in SG assembly. Reduction in the availability of mature mRNA in the cytoplasm can be achieved by limiting nascent RNA synthesis, inhibiting pre-mRNA splicing (52) or blocking mRNA export.

Although the role of newly transcribed RNA in SG formation was investigated previously through transcription inhibition, the effect of splicing inhibition or export had not. We examined the effects of various splicing inhibitors on the formation of RNA granules, focusing on SGs. A wide range of splicing inhibitors were applied, starting from inhibiting or knocking down SF3B1 (by PLB or siRNA) to using isoginkgetin that prevents the recruitment of U4/U5/U6 small nuclear RNPs (61), and madrasin that interferes with early stages of the spliceosome assembly, but does not target SF3B1 (44). A variety of splicing inhibitors led to a suppression in canonical SG assembly under stress conditions yet affected eIF2α phosphorylation differently. This supported the hypothesis that splicing inhibition has a negative effect on SG formation not via the phosphorylation of eIF2α, but due to a shortage of cytoplasmic mRNA, which in turn suppresses canonical SGs (Figures 2–5).

Intriguingly, we found that the splicing inhibitor madrasin leads to the formation of cytoplasmic assemblies, containing poly(A)^+^ RNAs and hallmark SG proteins G3BP1 and TIA1; however, other canonical SG proteins such as the translation initiation factors eIF3b, eIF4B and RPS6 were not localized to these granules (Figure 5 and Supplementary Figure S4). SGs that lack canonical proteins were described in previous studies and were categorized as ‘noncanonical SGs’. For instance, under conditions of chronic starvation, SGs lack RPS6 and RACK1, the latter being a protein that is associated with apoptosis signaling (62), suggesting that under these conditions the noncanonical SGs that form are associated with cell death (7). Therefore, we determined whether madrasin-induced granules are SGs. First, we showed that the assembly of these granules was not affected by pretreating the cells with ISRIB (Supplementary Figure S5C and D), which blocks SGs that are formed via the eIF2α phosphorylation mechanism (47,63). This also aligns with our finding that madrasin treatment does not elevate eIF2α phosphorylation levels (Figure 4D and E, and Supplementary Figure S5E and F). Second, cycloheximide treatment, which prevents polysome dissociation and is therefore used as a key experimental parameter for SG identification (64), did not affect madrasin-induced RNA granules (Supplementary Figure S5A and B). Altogether, these results propose that madrasin-induced RNA granules are not SGs *per se*. Adding arsenite to cells pretreated with madrasin led to the relocalization of eIF4B and eIF3b in SGs (Figure 5) (46,65). We suggest that madrasin treatment promotes the formation of a stress-like granule core, and then an additional stress (e.g. arsenite) induces the joining of additional RNBPs to the shell. Indeed, madrasin-induced granules seemed smaller than the canonical SGs induced by arsenite alone.

Observing the impact splicing inhibitors had on stress and stress-like RNA granules, it was interesting to determine whether the inhibition of splicing affects other RNA-containing granules, such as PBs and nuclear speckles. We found that the effects that the splicing inhibitors had on nuclear speckles and PBs were mechanism dependent; PLB, which targets SF3B1, increased PB size and numbers, unlike madrasin and isoginkgetin, which led to the loss of PBs, and did not re-form when arsenite was added (Figure 6 and Supplementary Figure S9). It is known that PB numbers increase during various stress conditions (66); therefore, we speculate that PLB treatment applies additional stress, leading eventually to increased PBs.

Since PLB showed a positive effect on PB abundance, and it is known that unspliced mRNAs cannot exit the nucleus, i.e. splicing inhibition leads to nuclear export inhibition, we examined whether inhibiting the export of mRNAs would lead to a similar effect on RNA granule assembly as stalling splicing. Indeed, blocking mRNA export stopped the formation of arsenite-induced SGs. However, PBs were not affected (Supplementary Figure S10A–C and Figure 7). We also observed that under splicing inhibition or export inhibition the quantity of mRNA in the cytoplasm decreased significantly (Supplementary Figure S10C and D, and Figure 7E). These findings point out that, first, shortage of newly synthesized mRNA in the cytoplasm is a limiting factor in SG formation. Second, these data imply that although mRNA is important for both PB and SG maintenance and assembly (50,66), they have different properties regarding the contribution of cytoplasmic mRNA to their formation. For instance, it was shown that yeast SGs cannot form without PBs, yet PBs can form without SGs. It is also known that PBs and SGs can dock and transfer components from one granule to another (67). This suggests that PBs are required for SG assembly. In addition, it was reported that loss of cytoplasmic RNA affects SGs and PBs differently—PBs were generally not affected, while SG assembly was inhibited (17). Altogether, we suggest that SGs are more sensitive to cytoplasmic mRNA depletion than PBs regarding their assembly, and this explains why no difference was observed in PBs in cells with export inhibition, while SG formation was clearly affected.

It was previously suggested that nascent mRNAs are essential for SG assembly due to a preferable characteristic as a newly exported mRNA (21). To understand whether export inhibition prevents the formation of SGs due to a lack of nascent transcripts, or due to a reduced number of cytoplasmic mRNAs, we synthesized synthetic mRNAs that were transfected directly to the cytoplasm. We show that transfection of *in vitro* transcribed mRNAs is sufficient for SG formation, and that mRNA quantity correlates with the population of cells positive for SGs (Figure 8A and B). It was previously demonstrated that an excess of mRNA promotes the formation of SGs. For example, one study showed that transfection of α-globin mRNA into puromycin-treated cells elevated SG assembly, indicating that an excess of nonpolysomal mRNA leads to SG formation (20,68). In a different study, it was demonstrated that purified cellular RNA forms assemblies *in vitro*, which have similar properties to SGs, and it was suggested that high concentration of RNA locally contributes to the assembly of the granule by RNA self-assembly (56). However, the underlying biological and molecular mechanism of the formation of these granules was not investigated yet. Here, we reveal that inhibiting PERK, which is activated during ER stress (69), or GCN2, which is associated with amino acid depletion (7), prior to transfection, blocked the formation of SGs induced by the *in vitro* transcribed mRNA (Figure 8C and D). Furthermore, nascent protein synthesis analysis and live-cell imaging revealed that these granules formed before proteins were synthesized (Figure 8E and F, and Supplementary Movies 5 and 6). Therefore, we conclude that although *in vitro* transcribed mRNA-induced SGs lead to the activation of signaling processes associated with ER stress and/or starvation, these granules are not directly formed due to ER stress. Further work is needed to reveal the detailed mechanism that connects these processes.

Finally, we wanted to determine whether transfecting mRNA into the cytoplasm that is relatively depleted from mRNAs due to splicing inhibition will allow the formation of arsenite-induced SGs. Indeed, treating the cells with isoginkgetin in combination with arsenite and *in vitro* transcribed mRNA transfection led to an elevation of cells that were positive for SGs (Figure 9A and B). Noteworthy, the population of cells that was positive for SGs under the latter conditions was significantly smaller than the population of cells positive for SGs under arsenite treatment alone. This may be explained by low variety in the cellular mRNAs, since RNA–RNA interactions are important in the assembly of SGs, or perhaps due to the lack of endogenously assembled RNBPs on the exogenous mRNAs (15,56). For instance, the contribution of certain mRNAs in the formation of SGs was demonstrated in a study that found untranslated mRNA transcripts that are essential for the formation of SGs (54).

Altogether, we demonstrate that newly transcribed untranslated RNA is important for the formation of SGs. Specifically, inhibition of mRNA transcription, splicing or export results in a shortage of nascent RNAs in the cytoplasm that limits SG formation. The abundance of the transcripts will determine the efficiency of SG assembly, and an excess of cytoplasmic mRNA can trigger SG formation and activate signaling pathways associated with protein synthesis and ER stress, even before translation initiation (Figure 9C). An abundance of cytoplasmic mRNA could partially rescue SG assembly even under splicing inhibition and stress conditions, pointing to the importance of having a cytoplasmic source of mRNA to initiate SG formation.

## Supplementary Material

gkae119_Supplemental_Files

## Data Availability

The datasets generated during and/or analyzed during the current study are available from the corresponding author on reasonable request.
