# Supplementary material for: Nuclear RNA-related processes modulate the assembly of cytoplasmic RNA granules
Source: Nucleic Acids Res. 2024 Feb 16;52(9):5356–75. doi: 10.1093/nar/gkae119 (PMC11109975; doi:10.1093/nar/gkae119)
Supplement: gkae119_Supplemental_Files [file gkae119_supplemental_files.zip › Supplementary Figures Angel et al revised.pdf]

Supplementary Figures

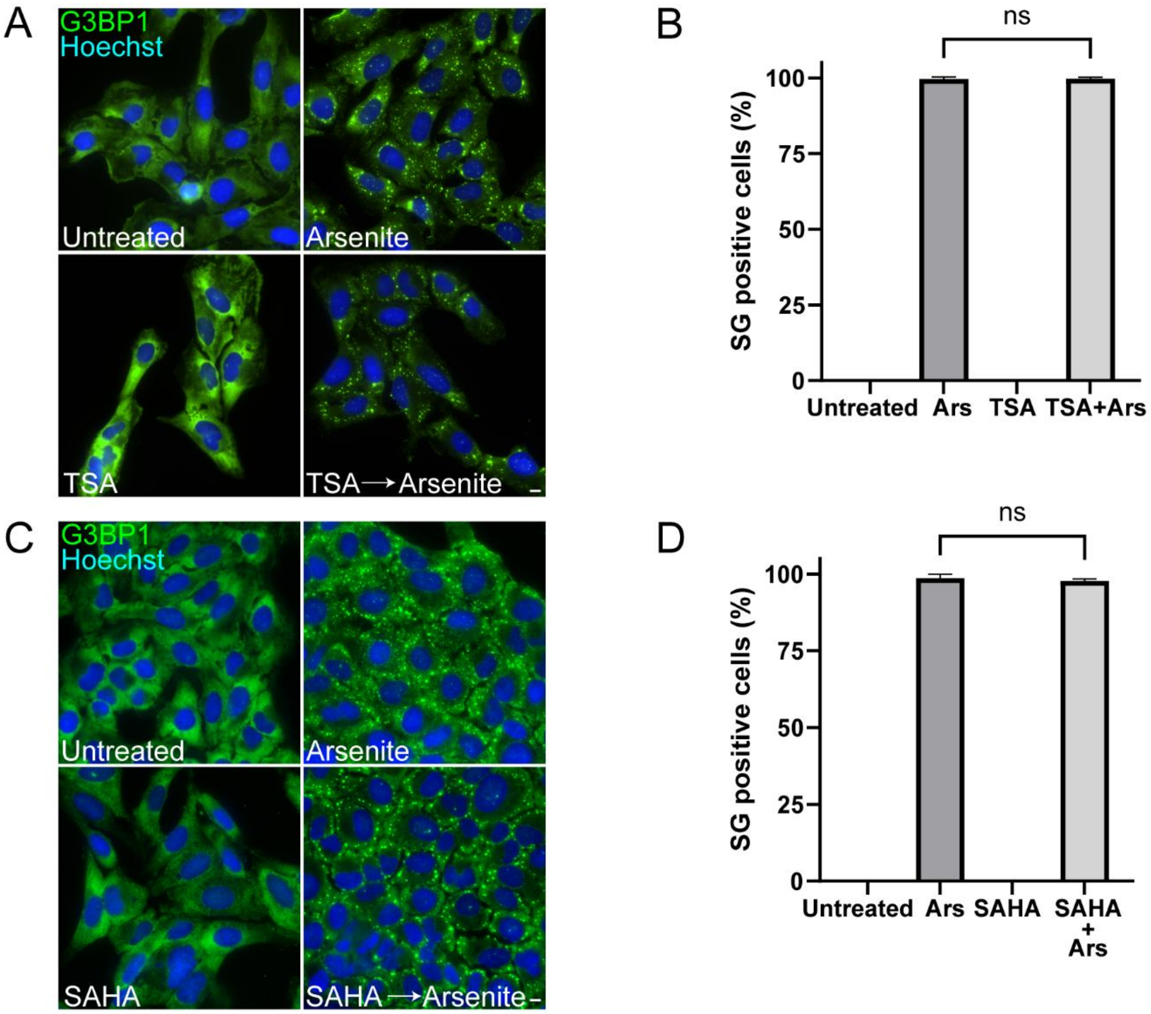

Supplementary Figure S1: Enhanced transcription does not affect SG formation. (**A**) U2OS cells were treated with TSA (150 nM) for 7 hrs, then arsenite (0.25 mM) was applied for 30 min. Cells were stained with anti-G3BP1 (green) to mark SGs. DNA stain is in blue. Scale bars= 10 µm. (**B**) Quantification of the population of SG-positive U2OS cells treated as described in A. Cells were counted in 3 independent experiments (n>300 cells per treatment). Data was analyzed with independent samples *t*-test (ns=non-significant). Bar graph illustrates the mean +/- standard deviation. (**C**) U2OS cells were treated with SAHA (150 ng/ml) for 24 hrs, then arsenite was applied for 30 min. Cells were stained with anti-G3BP1 (green) to mark SGs. DNA stain is in blue. (**D**) Quantification of the population of SG-positive U2OS cells treated as described in C. Cells were counted in 3 independent experiments (n>250 cells per treatment). Data was analyzed as described in B.

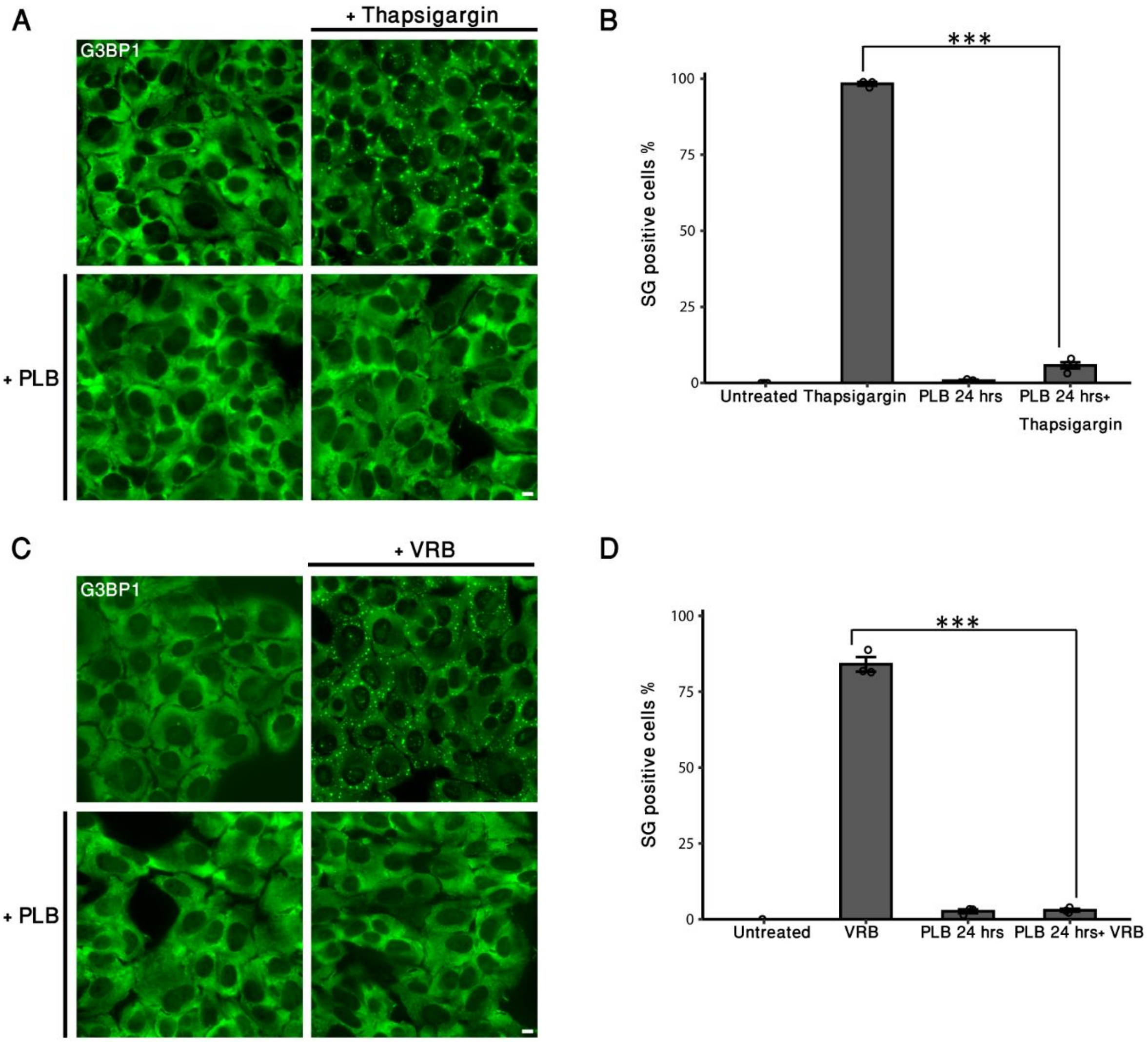
A
+ Thapsigargin
G3BP1
+ PLB
B
***
SG positive cells %
100
75
50
25
0
Untreated
Thapsigargin
PLB 24 hrs
PLB 24 hrs+ Thapsigargin
C
+ VRB
G3BP1
+ PLB
D
***
SG positive cells %
100
75
50
25
0
Untreated
VRB
PLB 24 hrs
PLB 24 hrs+ VRB

Supplementary Figure S2: The effect of splicing inhibition on SG formation is not limited to arsenite stress. (**A**) U2OS cells were treated with PLB (0.5 µM; 24 hrs) or with Thapsigargin (1 µM; 1 hr) and stained with anti-G3BP1 as a SG marker (green). (**B**) Quantification of the population of SG-positive U2OS cells treated as described in A. Cells were counted in 3 independent experiments (n>220 cells per treatment). Data were analyzed with independent sample *t*-tests (***$p<0.001$). Bar graph illustrates the mean +/- standard deviation. (**C**) U2OS cells were treated with PLB (0.5 µM; 24 hrs) and with VRB (100 µM; 1 hr), and stained with anti-G3BP1 (green). (**D**) Quantification of the population of SG-positive U2OS cells treated as described in C. Cells were counted in 3 independent experiments (n>140 cells per treatment). Data were analyzed as described in B. Scale bars= 10 µm.

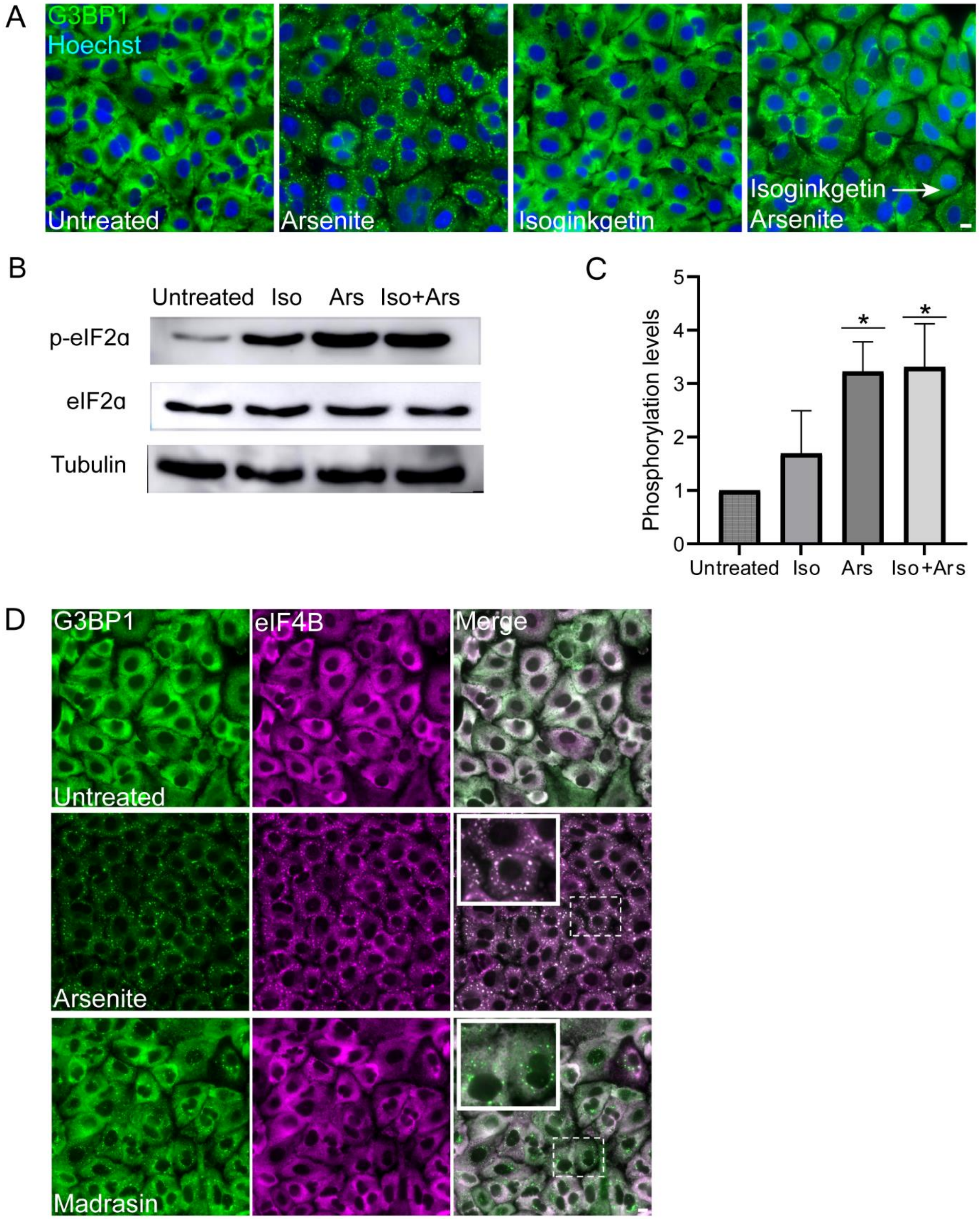
A
G3BP1
Hoechst
Untreated
Arsenite
Isoginkgetin
Isoginkgetin →
Arsenite
B
Untreated
Iso
Ars
Iso+Ars
p-eIF2α
eIF2α
Tubulin
C
Phosphorylation levels
0
1
2
3
4
5
*
*
Untreated
Iso
Ars
Iso+Ars
D
G3BP1
eIF4B
Merge
Untreated
Arsenite
Madrasin

Supplementary Figure S3: Splicing inhibitors affect SG formation induced by arsenite in A549 and U2OS cells. (**A**) A549 cells were treated with Isoginkgetin (100 µM; 4 hrs), and arsenite (0.25 mM) was added 30 min before fixation. Cells were stained with anti-G3BP1 as a SG marker (green). Hoechst DNA stain is in blue. (**B**) Western blot of protein extracts from U2OS cells treated with Isoginkgetin (Iso; 100 µM; 4 hrs) and/or with arsenite (Ars; 0.25 mM; 30 min). Blots were incubated with anti-eIF2α, anti-p-eIF2α, and anti-tubulin for loading control. (**C**) Quantifications of the eIF2α phosphorylation levels signal as seen in B. Phosphorylation signals in all samples were normalized to the total eIF2α protein level in each sample, and all samples were normalized to the untreated signal. Data were analyzed using ImageJ, and statistical analysis was carried out by a one-sample *t*-test against 0, to compare untreated to all other groups (n=3, *$p<0.05$). Bar graph illustrates the mean +/- standard deviation. (**D**) A549 cells were treated with madrasin (50 µM; 4 hrs) and with arsenite (0.25 mM) for 30 min before fixation. Cells were stained with anti-eIF4B (magenta) and anti-G3BP1 (green). SGs can be seen in the enlarged boxes. Scale bars= 10 µm.

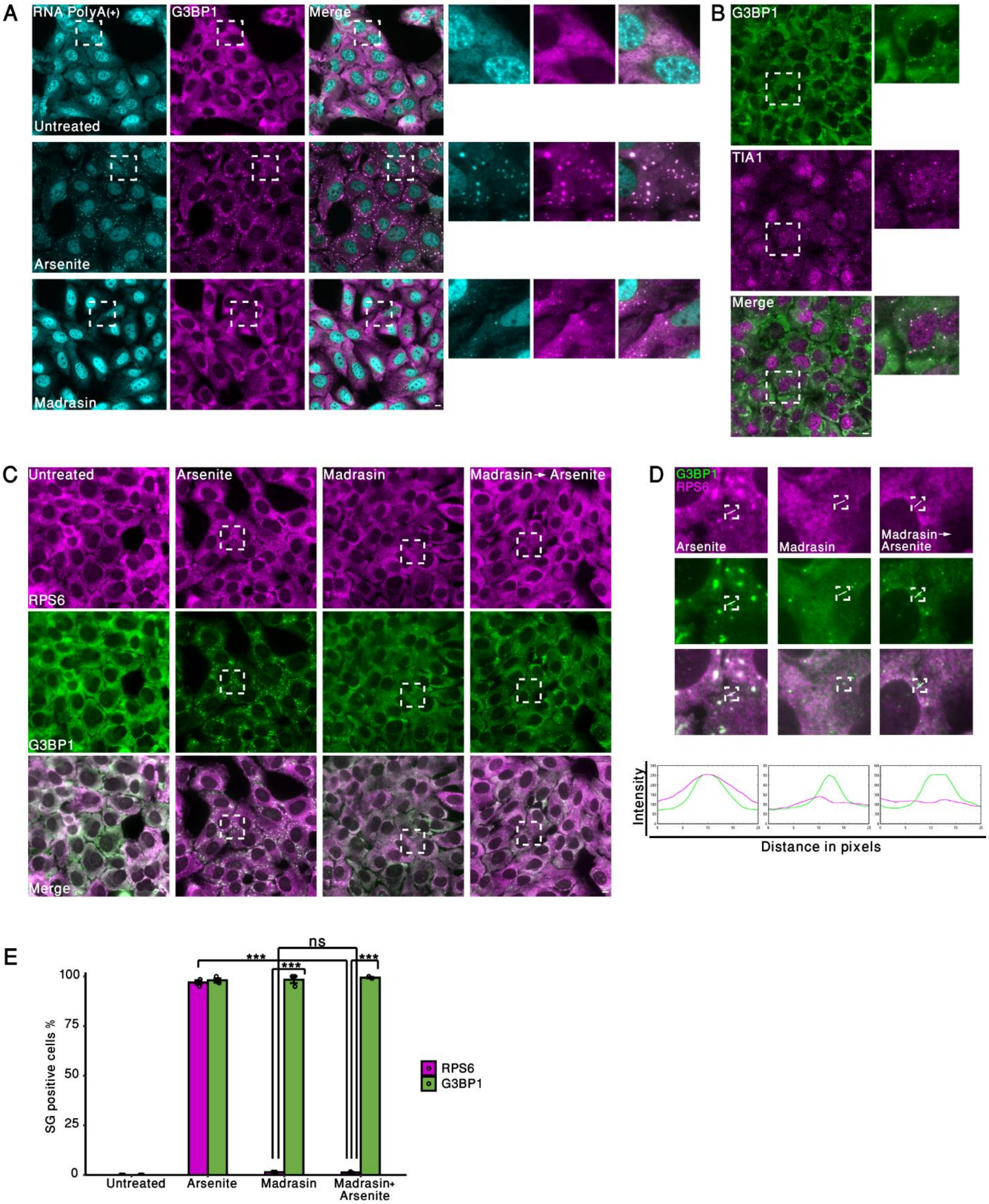
A
RNA PolyA(+)
G3BP1
Merge
Untreated
Arsenite
Madrasin
B
G3BP1
TIA1
Merge
C
Untreated
Arsenite
Madrasin
Madrasin→ Arsenite
RPS6
G3BP1
Merge
D
G3BP1
RPS6
Arsenite
Madrasin
Madrasin→ Arsenite
Intensity
Distance in pixels
E
ns
***
***
***
SG positive cells %
100
75
50
25
0
Untreated
Arsenite
Madrasin
Madrasin+ Arsenite
RPS6
G3BP1

Supplementary Figure S4: Madrasin induces stress-like granules. (**A**) U2OS cells were treated with madrasin (30 µM; 4 hrs), or with arsenite (0.25 mM; 30 min). Cells were stained by RNA FISH using a probe to poly(A)+ RNA (cyan) and by immunofluorescence with anti-G3BP1 (magenta). SGs in madrasin and arsenite treated cells can be seen in the enlarged boxes. (**B**) U2OS cells were treated with madrasin (30 µM; 4 hrs) and were stained with anti-G3BP1 (green) and anti-TIA1 (magenta) to mark SGs. (**C**) U2OS cells were treated with madrasin (30 µM; 4 hrs), and arsenite (0.25 mM) was added 30 min before fixation. Cells were stained with anti-RPS6 (magenta) and anti-G3BP1 (green). (**D**) Enlargements of designated areas are in the boxed regions from C. (Bottom) Intensity analysis from the boxed region along the white line. Scale bars= 10 µm. (**E**) Quantification of the population of SG-positive U2OS cells, marked by G3BP1 (green) or RPS6 (magenta), and treated as described in C. Cells were counted in 3 independent experiments (n>150 cells per treatment). Data were analyzed with a one-way ANOVA, followed by Tukey's post hoc analysis (****p*<0.001, ns=non-significant). Bar graph illustrates the mean +/- standard deviation.

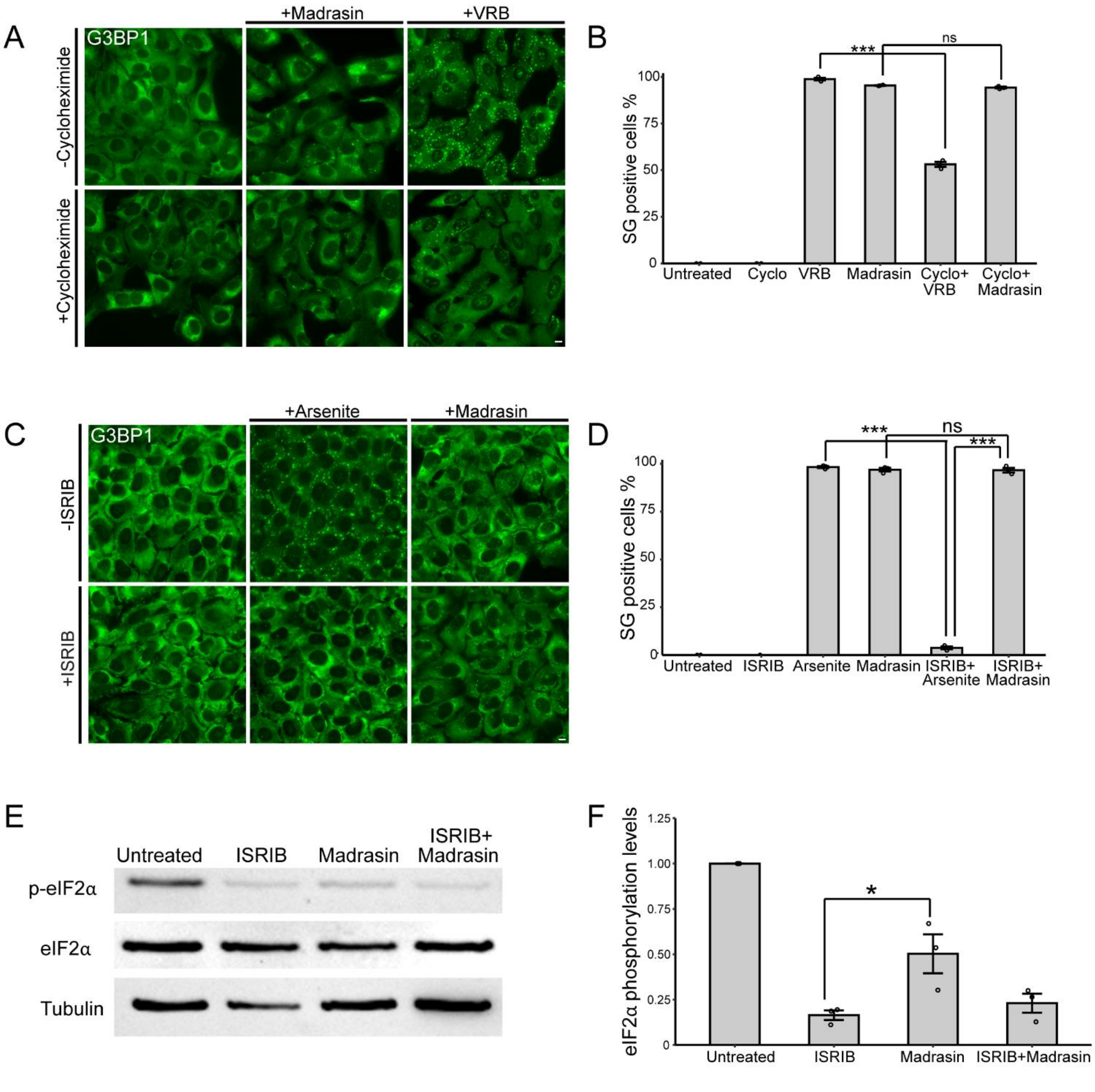
A
+Madrasin
+VRB
G3BP1
-Cycloheximide
+Cycloheximide
B
***
ns
SG positive cells %
100
75
50
25
0
Untreated
Cyclo
VRB
Madrasin
Cyclo+ VRB
Cyclo+ Madrasin
C
+Arsenite
+Madrasin
G3BP1
-ISRIB
+ISRIB
D
***
ns
***
SG positive cells %
100
75
50
25
0
Untreated
ISRIB
Arsenite
Madrasin
ISRIB+ Arsenite
ISRIB+ Madrasin
E
Untreated
ISRIB
Madrasin
ISRIB+ Madrasin
p-eIF2α
eIF2α
Tubulin
F
eIF2α phosphorylation levels
1.25
1.00
0.75
0.50
0.25
0
*
Untreated
ISRIB
Madrasin
ISRIB+Madrasin

Supplementary Figure S5: Madrasin-induced stress-like granules are not associated with classic SG-inducing pathways. (**A**) U2OS cells were treated with madrasin (30 μM; 4 hrs) or with VRB (100 μM; 1.5 hrs). Cycloheximide (100 μg/ml) was added 30 min before fixation, and cells were stained with anti-G3BP1 (green). Scale bars= 10 μm. (**B**) Quantification of the population of SG-positive U2OS cells treated as described in A. Cells were counted in 3 independent experiments (n>180 cells per treatment). Data were analyzed with one-way ANOVA, followed by Tukey's post hoc analysis (****p*<0.001, ns=non-significant). Bar graph illustrates the mean +/- standard deviation. (**C**) U2OS cells were treated with ISRIB (5 μM; 3 hrs), followed by madrasin (30 μM; 4 hrs) or with arsenite (0.25 mM; 30 min). Cells were stained with anti-G3BP1 as a SG marker (green). (**D**) Quantification of the population of SG-positive U2OS cells treated as described in C. Cells were counted in 3 independent experiments (n>200 cells per treatment). Data were analyzed as described in B, (n=3, ****p*<0.001, ns=non-significant). (**E**) Western blot of protein extracts from U2OS cells treated with ISRIB (5 μM; 3 hrs), followed by madrasin (30 μM; 4 hrs) or with arsenite (0.25 mM; 30 min). Blots were incubated with anti-eIF2α, anti-p-eIF2α and anti-tubulin for loading control. (**F**) Quantifications of the eIF2α phosphorylation levels signal as seen in E. Phosphorylation signals in all samples were normalized to the total eIF2α protein level in each sample, and all samples were normalized to the untreated signal, then data was log transformed. Data were analyzed using ImageJ, and statistical analysis was carried out by one-way ANOVA (n=3, **p*<0.05). Bar graph illustrates the mean +/- standard deviation.

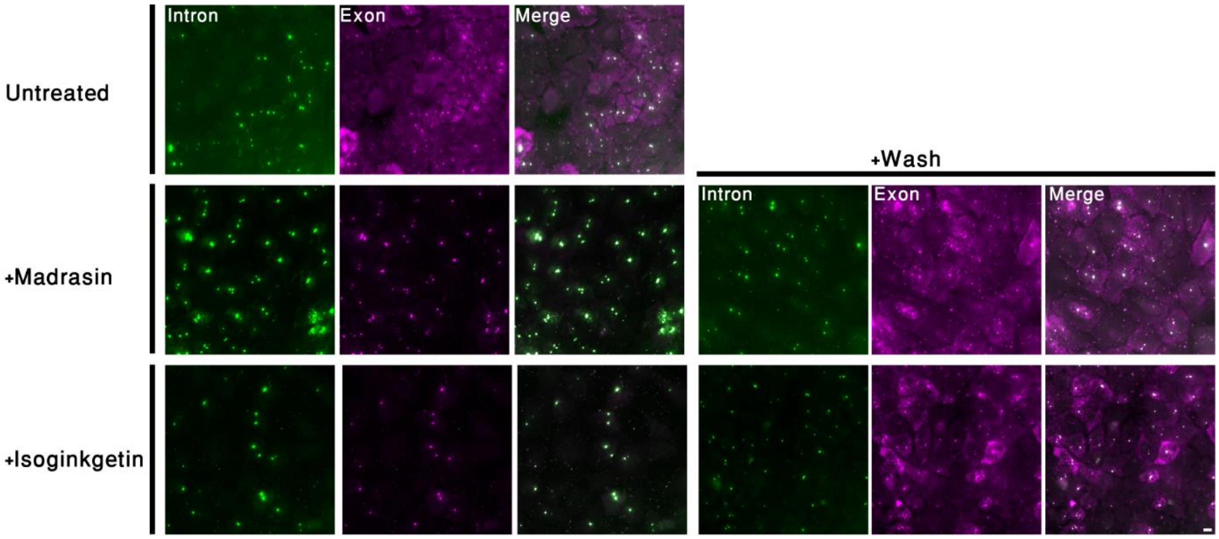

Supplementary Figure S6: Madrasin and Isoginkgetin inhibit splicing, and this effect is reversible. E6 cells were stained by RNA FISH using probes for the exon (magenta) and intron (green) of an inducible β-globin mini-gene expressed in E6 cells. Large dots are active transcription sites. Intron signal is usually seen on the active transcriptions sites while exon signal can also be observed in the cytoplasm. Cells were treated with madrasin (30 µM; 4 hrs) or Isoginkgetin (100 µM; 4 hrs), followed by a 24 hrs wash with fresh medium. Changes in the distribution of the exon signal limited to the nucleus can be seen during splicing inhibition, and release back to the cytoplasm upon wash. Scale bar= 10 µm.

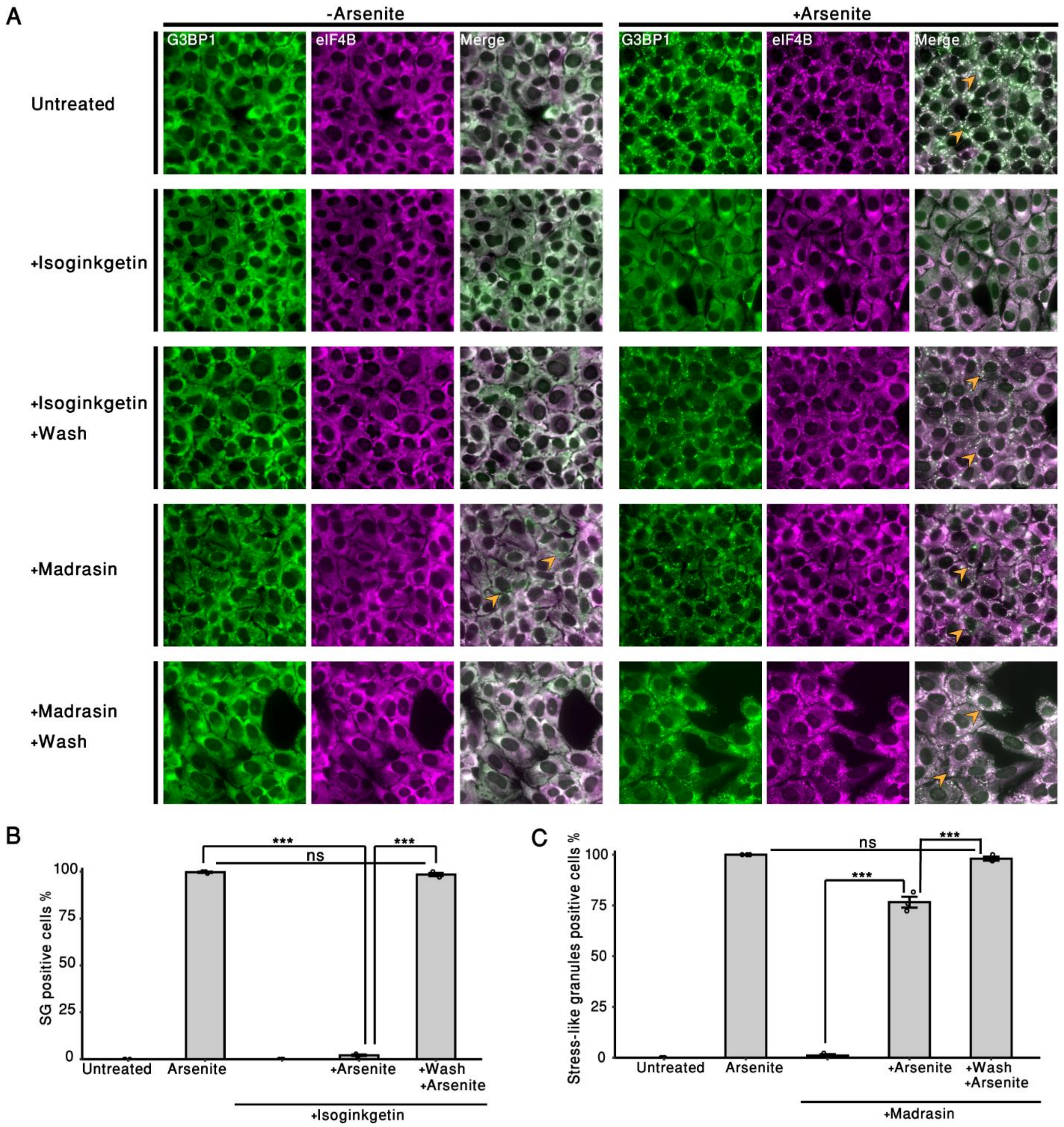
A
-Arsenite
+Arsenite
G3BP1
eIF4B
Merge
Untreated
+Isoginkgetin
+Isoginkgetin
+Wash
+Madrasin
+Madrasin
+Wash
B
SG positive cells %
***
ns
100
75
50
25
0
Untreated
Arsenite
+Arsenite
+Wash
+Arsenite
+Isoginkgetin
C
Stress-like granules positive cells %
Untreated
Arsenite
+Arsenite
+Wash
+Arsenite
+Madrasin

Supplementary Figure S7: Arsenite-induced SGs form after washing of splicing inhibitors. (**A**) U2OS cells were treated with Isoginkgetin (100 µM; 4 hrs) or madrasin (30 µM; 4 hrs). Cells were washed for 24 hrs with fresh medium, and arsenite (0.25 mM) was added 30 min before fixation. Staining was performed using anti-G3BP1 (green) and anti-eIF4B (magenta) as SG markers. Scale bar= 10 µm. Orange arrowheads point to SG-positive cells. (**B**) Quantification of the population of SG-positive U2OS cells treated as described in A. Cells positive for SGs were counted using the G3BP1 marker in 3 independent experiments (n>250 cells per treatment). Data were analyzed with one-way ANOVA, followed by Tukey's post hoc analysis (***p<0.001, n=non-significant). Bar graph illustrates the mean +/- standard deviation. (**C**) Quantification of the population of SG-positive U2OS cells treated as described in A. Cells positive for stress-like granules were counted using the eIF4B marker in 3 independent experiments (n>180 cells per treatment). Data were analyzed as described in B (***p<0.001, n=non-significant). Bar graph illustrates the mean +/- standard deviation.

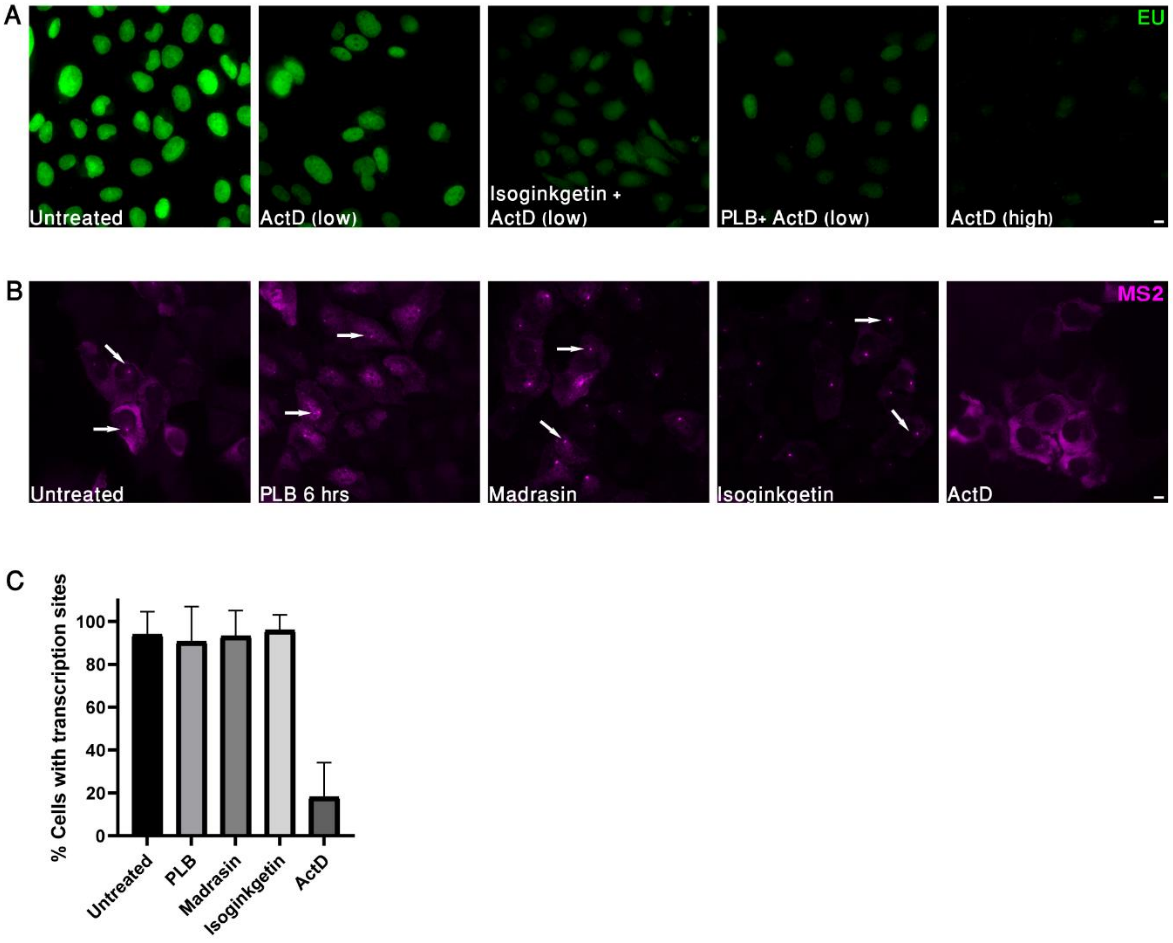

Supplementary Figure S8: The effect of splicing inhibitors on transcription. (**A**) U2OS cells were treated with Isoginkgetin (100 µM; 4 hrs), PLB (0.5 µM; 6 hrs), or ActD (5 µg/ml; 2 hrs). ActD at a lower concentration (0.05 µg/ml) was applied 2 hrs before fixation to eliminate rRNA synthesis by RNA polymerase I and nucleolar staining. Nascent RNAs were labeled using click-it EU (green). (**B**) E3 cells expressing an inducible β-globin minigene containing MS2 sequence repeats cells were treated with Isoginkgetin (100 µM; 4 hrs), PLB (0.5 µM; 6 hrs), madrasin (30µM; 4 hrs) or ActD (5 µg/ml; 2 hrs). Cells were stained by RNA FISH using a probe to the MS2 sequences and active transcription sites were detected. Scales bars= 10 µm. (**C**) Quantifications of transcription sites in U2OS cells treated as described in B (n>40 cells per treatment, 3 independent experiments).

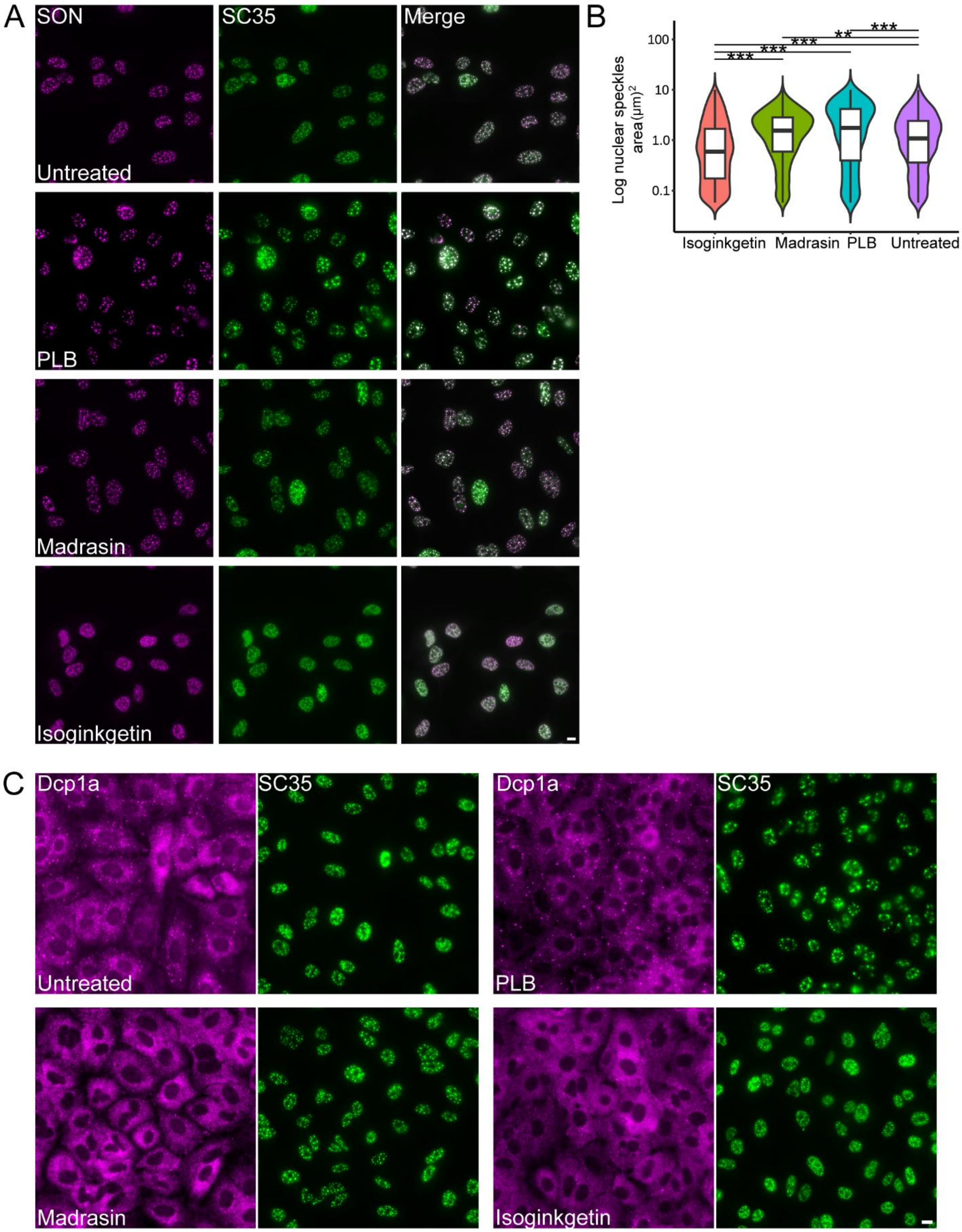
A
SON
SC35
Merge
Untreated
PLB
Madrasin
Isoginkgetin
B
Log nuclear speckles area (μm)^2
100
10
1.0
0.1
***
***
***
**
***
Isoginkgetin
Madrasin
PLB
Untreated
C
Dcp1a
SC35
Dcp1a
SC35
Untreated
PLB
Madrasin
Isoginkgetin

Supplementary Figure S9: The effect of splicing inhibitors on nuclear speckles and P bodies. (**A**) U2OS cells were treated with PLB (0.5 µM; 6 hrs), madrasin (30 µM; 4 hrs), and with Isoginkgetin (100 µM; 4 hrs). Cells were stained with anti-SON (magenta) and anti-SC35 (green) as markers of nuclear speckles. Scale bars= 10 µm. (**B**) Violin plot representing the area (log) of nuclear speckles, that were stained using anti-SON, in cells treated as described in A. Data were analyzed using one-way nested ANOVA, followed by Tukey's post hoc analysis. More than 1,000 nuclear speckles were quantified for each experiment per treatment (n=3). The graph represents one experiment, and the statistical test for significance was carried for all 3 independent experiments (**p<0.01, ***p<0.001). (**C**) A549 cells were treated with PLB (0.5 µM; 6 hrs), madrasin (50 µM; 4 hrs), and with Isoginkgetin (100 µM; 4 hrs). Arsenite (0.25 mM) was added 30 min before fixation, and cells were stained with anti-Dcp1a (magenta) as a PB marker and anti-SC35 as a nuclear speckles marker (green).

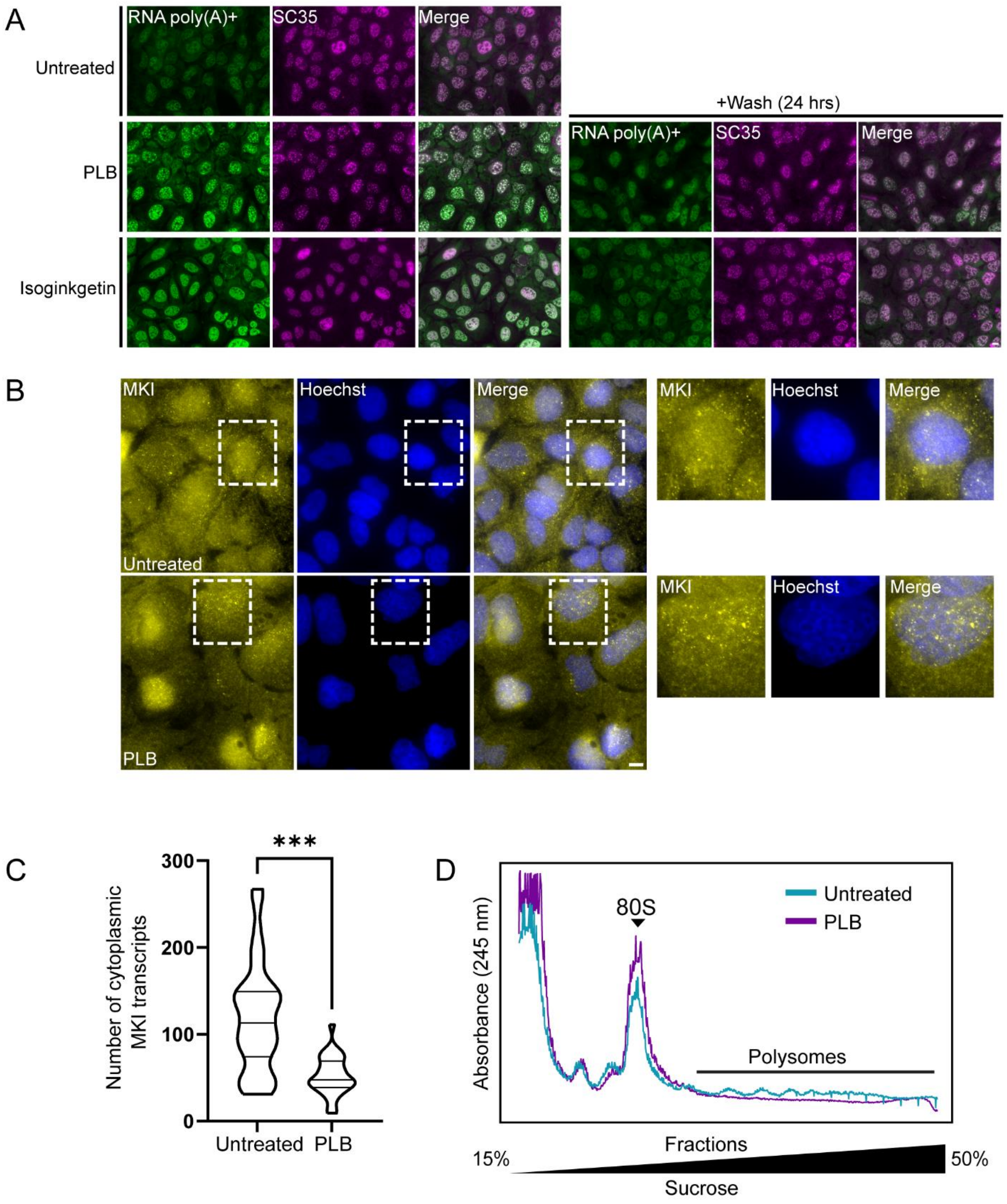
A
RNA poly(A)+
SC35
Merge
Untreated
PLB
Isoginkgetin
+Wash (24 hrs)
B
MKI
Hoechst
Merge
Untreated
PLB
C
***
Number of cytoplasmic MKI transcripts
300
200
100
0
Untreated
PLB
D
80S
Untreated
PLB
Absorbance (245 nm)
Polysomes
Fractions
15%
50%
Sucrose

Supplementary Figure S10: Splicing inhibition blocks export of poly(A)+ transcripts. (**A**) U2OS cells were treated with PLB (0.5 µM; 6 hrs) or with Isoginkgetin (100 µM; 4 hrs) and washed with fresh medium for 24 hrs. Cells were stained by RNA FISH using a fluorescent probe to poly(A)+ RNA (green) and immunofluorescence with anti-SC35 (magenta) as a marker of nuclear speckles. The increase in nuclear poly(A)+ signal indicates an mRNA export block. Scale bars=10 µm. (**B**) RNA smFISH targeting the MKI mRNA (yellow) in untreated and PLB (0.5 µM, overnight) treated U2OS cells. Hoechst DNA staining (blue). (Right) Enlargements of the marked boxes. (**C**) Truncated violin plot representing the number of cytoplasmic MKI transcripts in U2OS cells, as treated in A. Data were quantified using Imaris and the statistical analysis was carried out using unpaired *t*-test (***$p<0.001$, n=30 cells per treatment). (**D**) Polysome profile for U2OS control cells (untreated) and PLB treated (0.5 µM, overnight). Polysome profiles were performed using 15–50% sucrose density gradients, and the position of the 80S monosome and the polysomes are indicated.

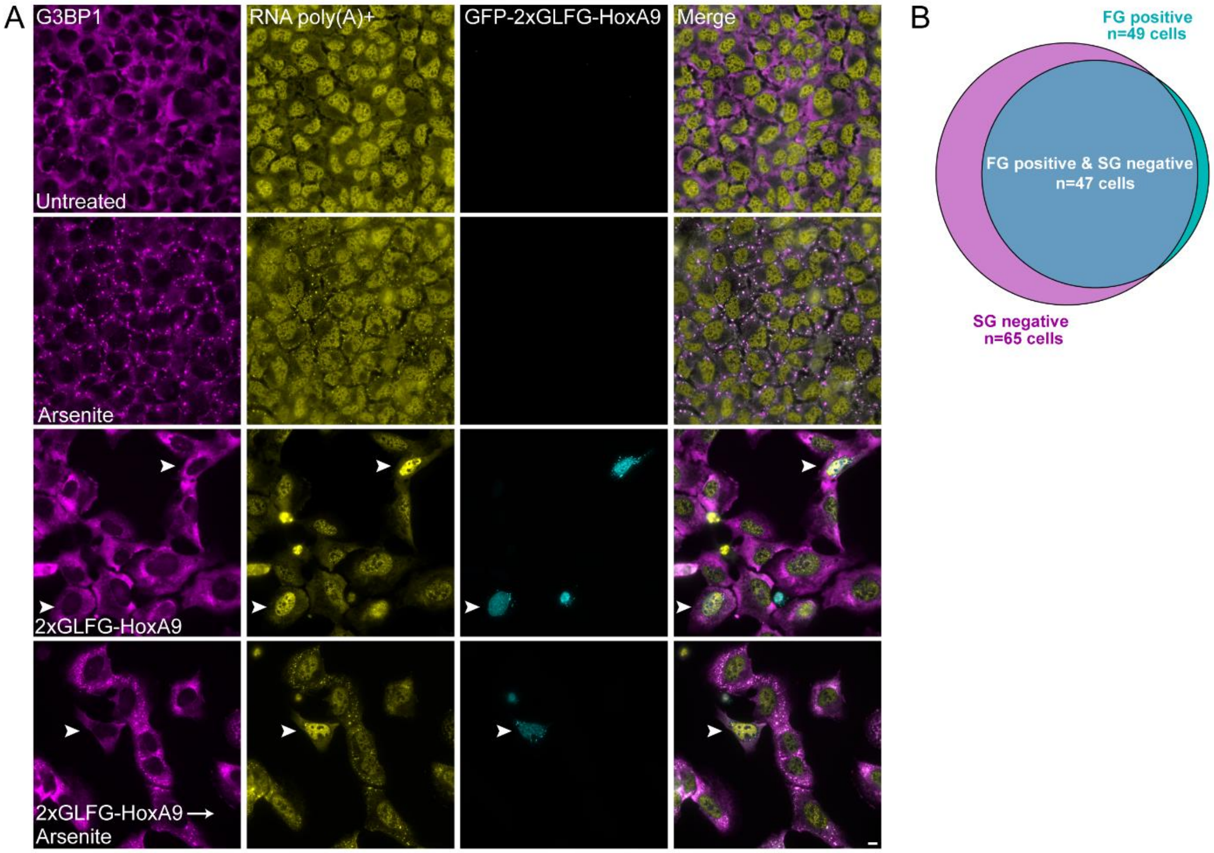

Supplementary Figure S11: The effect of export inhibition on cytoplasmic RNA granules. (**A**) U2OS cells were transfected with GFP-2XGLFG-HoxA9 (FG repeats; 24 hrs) to block mRNA export, following arsenite treatment (0.25 mM, 30 min) prior to fixation. After cells were transfected with GFP-2XGLFG-HoxA9 (cyan), they were stained by RNA FISH using a fluorescent probe to poly(A)+ RNA (yellow), followed by immunofluorescence with anti-G3BP1 as a marker for SGs (magenta). White arrowheads point to positive-transfected cells. Scale bar=10 µm. (**B**) Venn diagram showing the population of SG positive cells and export inhibited cells (i.e., 2XGLFG-HoxA9 positive cells). U2OS cells were treated as described in B (n=3).

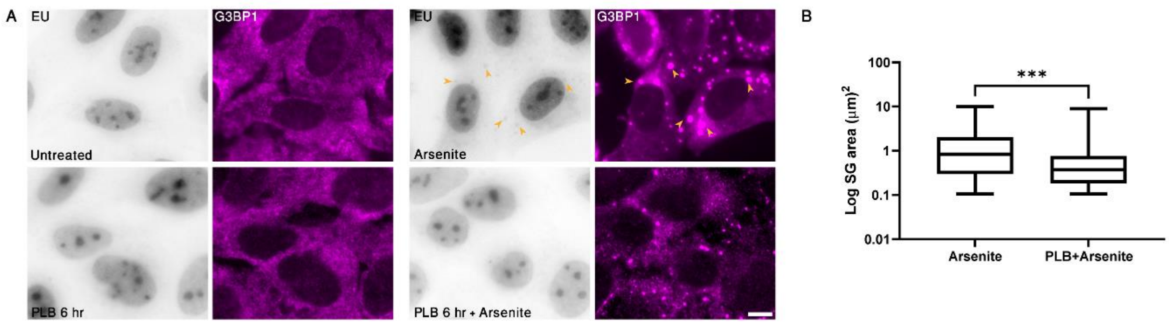

Supplementary Figure S12: Nascent RNA transcripts are excluded from SGs induced by arsenite when splicing is inhibited. (**A**) U2OS cells were treated with PLB (0.5 µM; 6 hrs), and arsenite (0.25 mM) was added 1 hr before fixation. Nascent RNAs were labeled using click-it EU for 2 hrs (inverted) and immunoassayed with anti-G3BP1 (magenta) to mark SGs. Orange arrows point to SGs. Scale bar= 10 µm. (**B**) Box plot representing the area of SG, that were stained using anti-G3BP1, in cells treated as described in A. Data were analyzed using independent *t*-test. More than 4,500 SGs were quantified per treatment (****p*<0.001, n=3).

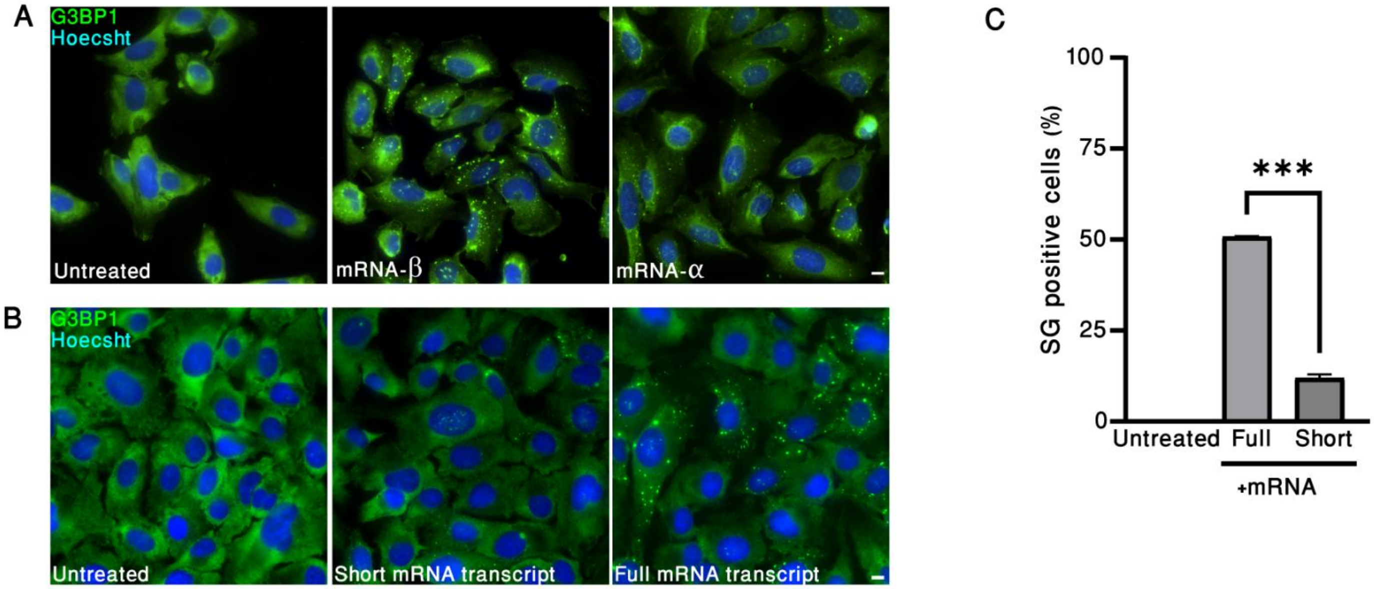

Supplementary Figure S13: Delivery of mRNA into the cytoplasm induces SG formation. (**A**) U2OS cells were transfected with TCR α and β chains mRNA transcripts (1 μg/ml) for 1.5 hrs. Cells were stained with anti-G3BP1 (green) to mark SGs. DNA Hoechst stain is in blue. (**B)** U2OS cells were transfected with full length or shortened GFP mRNAs (0.6 μg/ml) for 1.5 hrs. Cells were stained with anti-G3BP1 (green) to mark SGs. DNA Hoechst stain is in blue. Scale bars= 10 μm. (**C**) Quantification of the population of SG-positive U2OS cells treated as described in B. Cells were counted in 3 independent experiments (n>220 cells per treatment). Data were log transformed and were analyzed with independent samples *t*-test (****p*<0.001). Bar graph illustrates the mean +/- standard deviation.

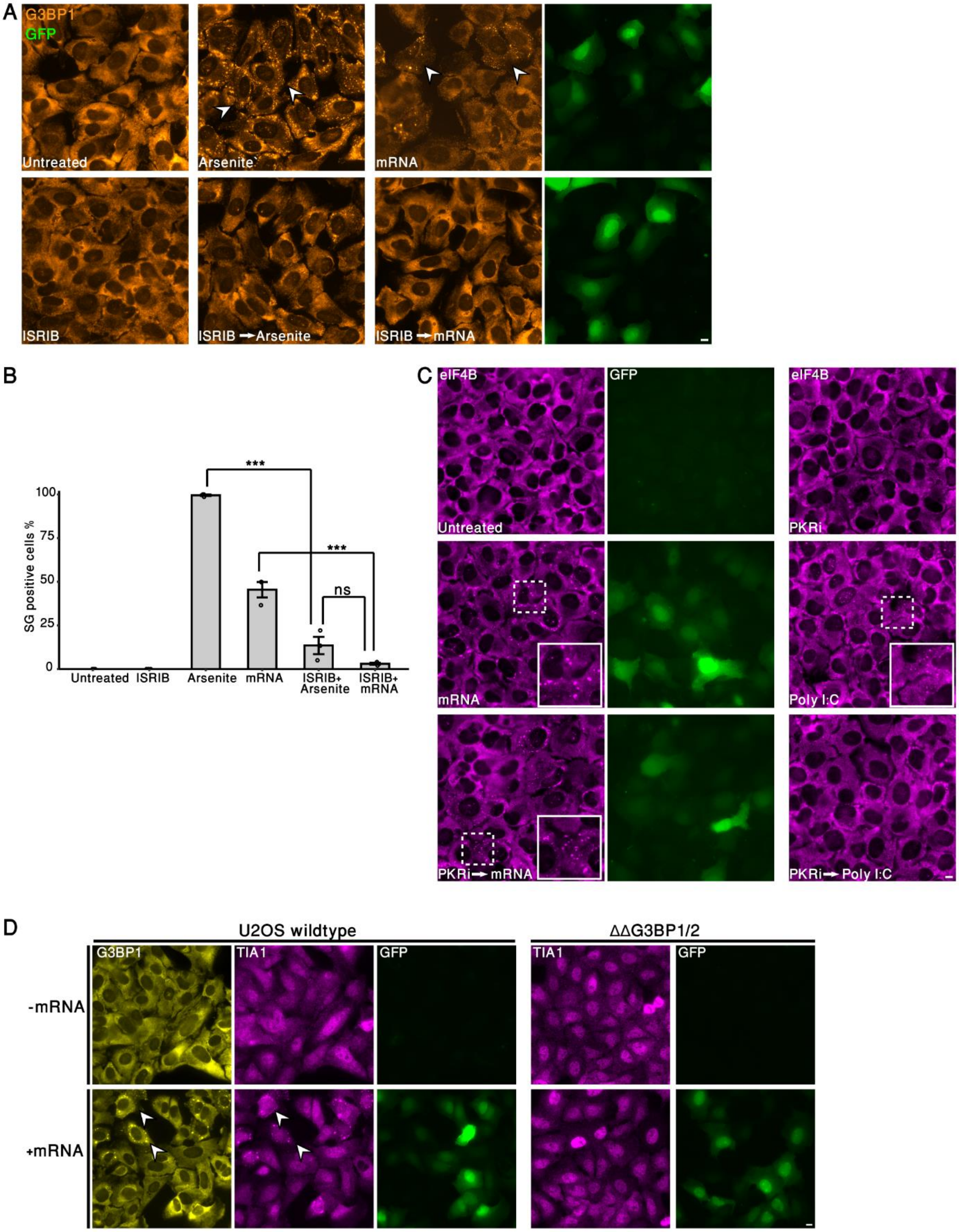
A
G3BP1
GFP
Untreated
Arsenite`
mRNA
ISRIB
ISRIB → Arsenite
ISRIB → mRNA
B
SG positive cells %
100
75
50
25
0
***
***
ns
Untreated
ISRIB
Arsenite
mRNA
ISRIB+ Arsenite
ISRIB+ mRNA
C
eIF4B
GFP
eIF4B
Untreated
PKRi
mRNA
Poly I:C
PKRi → mRNA
PKRi → Poly I:C
D
U2OS wildtype
ΔΔG3BP1/2
G3BP1
TIA1
GFP
TIA1
GFP
-mRNA
+mRNA

Supplementary Figure S14: Synthetic mRNA induces the formation of canonical SGs. (**A**) U2OS cells were treated with ISRIB (5 µM) for 4.5 hrs and with arsenite (0.25 mM) for 30 min. *In vitro* transcribed mRNA (0.5 µg) encoding GFP (green) was transfected using Lipofectamine, and cells were fixed 1.5 hrs after transfection. Cells were stained with anti-G3BP1 (orange) as a SG marker. White arrowheads point to SG-positive cells. (**B**) Quantification of the population of SG-positive U2OS cells treated as described in A. Cells were counted in 3 independent experiments (n>200 cells per treatment). Data were analyzed with one-way ANOVA, followed by Tukey's post hoc analysis (***p<0.001, n=non-significant). Bar graph illustrates the mean +/- standard deviation. (**C**) U2OS cells were treated with a PKR inhibitor (0.4 µM) for 3 hrs, then transfected with mRNA (1 µg) encoding GFP (green) for 1.5 hrs. Cells were stained with anti-eIF4B (magenta). Poly(I:C) (1 µg/ml; 4 hrs) was used as a positive control. SGs can be seen in the enlarged boxes. (**D**) U2OS wildtype and U2OS ΔΔG3BP1/2 cells were transfected with mRNA (1 µg) encoding GFP (green) for 1.5 hrs. Cells were stained with anti-G3BP1 (yellow) and anti-TIA1 (magenta). White arrowheads point to SG-positive cells. Scale bars= 10 µm.

Movie legends

Movie 1: Live-cell imaging of U2OS cells stably expressing GFP-IGF2BP3 without treatment. Images were acquired every 3 minutes for 39 minutes. Scale bar=10 µm.

Movie 2: Live-cell imaging of U2OS cells stably expressing GFP-IGF2BP3 pre-treated with Isoginkgetin (100 µM; 4 hrs). Then images were acquired every 3 minutes for 39 minutes. Scale bar=10 µm.

Movie 3: Live-cell imaging of U2OS cells stably expressing GFP-IGF2BP3 with arsenite (0.25 mM) treatment. Images were acquired every 3 minutes for 39 minutes. Scale bar=10 µm.

Movie 4: Live-cell imaging of U2OS cells stably expressing GFP-IGF2BP3 pre-treated with Isoginkgetin (100 µM; 4 hrs), followed by by the addition of arsenite (0.25 mM) at the starting time point (t=0 min). Images were acquired every 3 minutes for 39 minutes. Scale bar=10 µm.

Movie 5: Live-cell imaging of U2OS cells stably expressing the SG marker protein mCherry-TIA1 transfected with mRNA (1 µg) encoding for the protein GFP. Images were acquired every 10 minutes for 300 minutes. Scale bar=10 µm.

Movie 6: Live-cell imaging of GFP expression in U2OS cells stably expressing mCherry-TIA1 as shown in movie 5, transfected with mRNA (1 µg) encoding for GFP. Images were acquired every 10 minutes for 300 minutes. Scale bar=10 µm.
